# Molecular pathophysiology of diabetes mellitus during pregnancy with antenatal complications

**DOI:** 10.1038/s41598-020-76689-9

**Published:** 2020-11-12

**Authors:** Arthur T. Kopylov, Olga Papysheva, Iveta Gribova, Galina Kotaysch, Lubov Kharitonova, Tatiana Mayatskaya, Ekaterina Sokerina, Anna L. Kaysheva, Sergey G. Morozov

**Affiliations:** 1 Department of Pathology, Institute of General Pathology and Pathophysiology, 8 Baltyiskaya str., 125315 Moscow, Russia; 2 Institute of Biomedical Chemistry, Biobanking Group, 10 Pogodinskaya str., 119121 Moscow, Russia; 3 S.S. Yudin 7th State Clinical Hospital, 4 Kolomenskaya str., 115446 Moscow, Russia; 4 N.E. Bauman 29th State Clinical Hospital, 2 Hospitalnaya sq., 110020 Moscow, Russia; 5 N.I. Pirogov Medical University, 1 Ostrovityanova st., 117997 Moscow, Russia

**Keywords:** Proteomics, Systems biology, Endocrine system and metabolic diseases, Metabolic disorders, Endocrine system and metabolic diseases, Molecular medicine, Hormones, Lipids

## Abstract

Gestational diabetes mellitus is a daunting problem accompanied by severe fetal development complications and type 2 diabetes mellitus in postpartum. Diagnosis of diabetic conditions occurs only in the second trimester, while associated antenatal complications are typically revealed even later. We acquired an assay of peripheral and cord blood samples of patients with different types of diabetes mellitus who delivered either healthy newborns or associated with fetopathy complications. Obtained data were handled with qualitative and quantitative analysis. Pathways of molecular events involved in diabetes mellitus and fetopathy were reconstructed based on the discovered markers and their quantitative alteration. Plenty of pathways were integrated to differentiate the type of diabetes and to recognize the impact of the diabetic condition on fetal development. The impaired triglycerides transport, glucose uptake, and consequent insulin resistance are mostly affected by faulted lipid metabolism (*APOM*, *APOD*, *APOH*, *APOC1*) and encouraged by oxidative stress (*CP*, *TF*, *ORM2*) and inflammation (*CFH*, *CFB*, *CLU*) as a secondary response accompanied by changes in matrix architecture (*AFM*, *FBLN1*, *AMBP*). Alterations in proteomes of peripheral and cord blood were expectedly unequal. Both up- and downregulated markers were accommodated in the cast of molecular events interconnected with the lipid metabolism, RXR/PPAR-signaling pathway, and extracellular architecture modulation. The obtained results congregate numerous biological processes to molecular events that underline diabetes during gestation and uncover some critical aspects affecting fetal growth and development.

## Introduction

Gestational diabetes mellitus (GDM) is a form of hyperglycemia, exhibited as an impairment of glucose tolerance, which first occurred during gestation and accounted for the majority of diabetes in pregnancies. It is possible that the disorder could precede pregnancy but has not been indicated previously^1^. The main reason for GDM is a progressive increase in insulin resistance in the course of gestation, caused by both placental and maternal hormones (prolactin, estrogen, and cortisol). Elevating concentration of these hormones is balanced by decreasing endogenous insulin clearance, but the condition is aggravated by calories-rich food, low mobility, and increased own body weight during gestation. As gestation progresses, insulin steadily decreases, leading to its large concentration due to compensatory mechanism activation. Obesity and hereditary propensity for diabetes type 2 diabetes mellitus are major endogenous risk factors. Patients with polycystic ovary syndrome and arterial hypertension are also in the group of the increased GDM risk. The age factors, overweight, and signs of GDM over previous gestations were also reported as master risk factors for GDM^2–4^.

The GDM is accompanied by various obstetrical and perinatal complications, among which diabetic fetopathy (DF) is the most common form and reason for possible perinatal loss^5,6^. In turn, the DF frequently causes premature birth, asphyxia of fetus, metabolic disorders, a complication in the adaptation of newborn and its death^6,7^. In most observed cases, the GDM does not manifest itself as any morphological changes in the placenta. On occasion, atherosclerotic vascular damage can be observed; however, this indicator can also be found if chronic decompensation of diabetes is inspected^8^.

Unfortunately, today there are no strong and definite criteria for diagnostic of GDM in the early course of gestation. Since GDM commonly encourages asymptomatic^9^, typically, GDM is detected during the prenatal screening^4^ rather than for the reason of reported symptoms. Recommendations of the International Federation of Gynecology and Obstetrics (IFGO)^10^ and IDF^6^ include the application of oral glucose tolerance test (OGTT) in 24–28 weeks of pregnancy as the most accurate method for the diagnosis of GDM.

One of the major approaches for the treatment of diabetic conditions during pregnancy and for the prevention of dire consequences of fetus development is pharmacological management. It is generally limited to the administration of insulin to normalized maternal blood glucose levels. The injection of insulin typically occurs subcutaneously, which creates a barrier for its sufficient utilization. That is why women with gestational diabetes are also treated with oral antidiabetic agents. Another conventional approach provides therapy by administration with insulin secretagogues like tolbutamide, chlorpropamide, Glucotrol, glyburide, glibenclamide, etc.^11^. In this case, it is expected that these compounds have to stimulate insulin secretion if they may cross the placenta barrier. Obviously, this can make the already observed diabetic fetopathy worse, albeit at the cost of lowering the circulating glucose levels.

Dietary intervention accompanied by the strict monitoring of lifestyle, especially in early pregnancy, is also the most popular approach for the prevention of GDM or management of T2DM and aimed to conduct the conditions to prevent diabetes fetopathy. Notwithstanding, recent reports showed that despite the extensive intervention of the Diabetes Prevention Program and Diabetes Prevention Study, almost 15% of women still had GDM^12^. Improving the lifestyle before pregnancy and postpartum gives insignificantly better results compare to dietary intervention^13^. The main issue of lifestyle improvement and diet management focuses on the first-trimester time-points when methods for diagnosis of GDM and DF are still insensitive. Currently, actual clinical diagnostics can provide opportunities from 22–24 and typically from the 30 gestational weeks to establish GDM and DF, correspondingly.

Both approaches usually demonstrate equal efficiency in the treatment of GDM and prevention of diabetic fetopathy and depict unsuccessful rate outcome of almost 18% according to the WHO annual report on diabetes mellitus and its complication for the 2018 year. So, it is not easy to estimate the effectiveness of any approaches due to the wasted time caused by insufficient OGTT and instrumental sensitivity. Due treatment is also challenged by the accompanied accidences of equivocal diagnosis when once it has been established, fetal hyperinsulinemia may contribute to an exaggerated fetal glucose steal^5^, which can explain the occurrence of macrosomia in pregnancies with normal maternal glucose values in late pregnancy.

Currently, sonography examination is the most confident way for diagnosis and monitoring of DF. During the second and third trimesters, the dynamic sonography examination can register an advanced fetus development progression as a consequence of hyperinsulinemia^14^. In the case of the occasion, the sonography examination displays an increased fetus size by two weeks ahead of the actual period, edematous and disproportion of the developing fetus. However, the limitation of the actually employed instrumental methods for diagnosing antenatal complications is the inability to reveal confident features of DF in the first trimester. The primary reason for insufficient sensitivity is the lack of reliable clinical markers that would permit diagnosis and support estimation of the possible risk of DF at diabetic condition over the entire gestation course.

The primary goal of this study is a proteome-scaled determination of unique markers and their combination in peripheral and umbilical blood to embed them into the reconstructed chain of molecular events for metabolic impairments during gestation and possible influence on fetal development.

## Results

**Figure 1:**
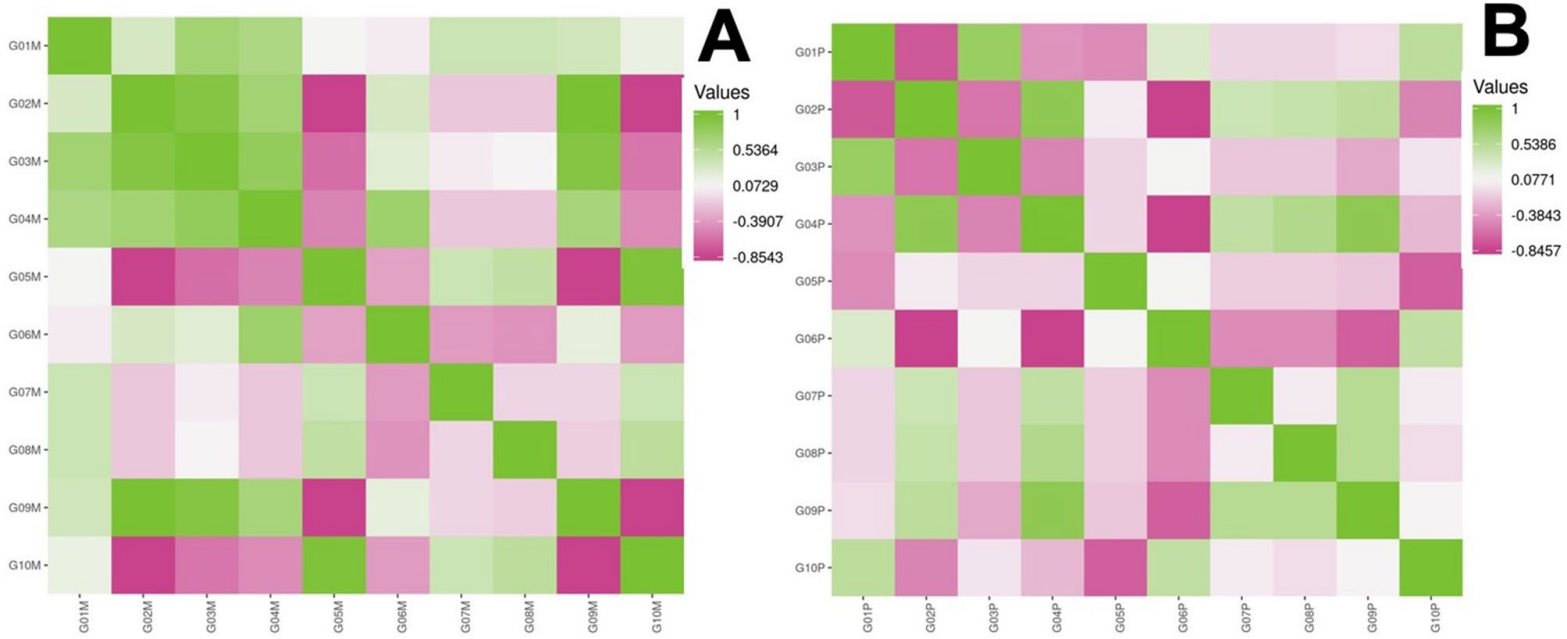
Matrix of pairwise Kendall' s-*tau* rank correlation between the studied groups. The Kendall-tau correlation coefficient can take values from -1 to 1, where the most negative value reflects a negative correlation between weighted parameters. In contrast, the most positive values reflect positive intergroup correlation. The obtained Kendall’s-*tau* correlation coefficient in the M-series **(A)** of peripheral blood samples ranged between − 0.757 (pair of G07M and G06M groups) to 0.938 (between G09M and G02M groups). In the C-series **(B)** of umbilical blood samples the studied groups the Kendall’s-*tau* correlation touched between − 0.554 (pair of G06P and G02P groups) and 0.896 (pair of G07P and G09P groups).

The study population comprised 264 patients stratified according to the established type of diabetes mellitus and its antenatal complication observed during the routine clinical screening in the course of gestation (Table 1). Patients with different diabetic conditions (GDM, T1DM, and T2DM) did not distinguish significantly in their BMI (p = 0.839) but differed from the obesity group (G06, p = 0.007) and, expectedly, from the control group (G05, p = 0.003). Since samples were collected between 23–28 weeks of the gestational age across all studied groups, there was no difference in the results of OGTT for the groups of GDM positive patients (G01-G04, p = 0.871). The results of fasting glucose levels between patients with T1DM and T2DM were almost indistinguishable (G07-G10, p = 0.172) but considerably higher than the test results on the GDM positive groups (p = 0.0217). Glycated hemoglobin value displayed mild increasing tendency during gestation, which indicates stable insulin resistance in patients irrespective of the type of diabetes mellitus. Patients with a different strategy of GDM management (insulin treatment or dietary intervention, Table 1 and Fig. 1) also did not demonstrate the meaningful difference in HbA1c value (p = 0.655).

**Table 1:** Main anthropometric data and measurements of glucose level for patients in study groups.

Group ID	iGDM^★^		dGDM ^∇^		Control	Obesity^¶^	T1DM		T2DM		p-value
Groups description	G01	G02	G03	G04	G05	G06	G07	G08	G09	G10	
Ultrasound examination^†^	NC	DF	NC	DF	NC	NC	DF	NC	DF	NC	
Groups size	30	25	26	29	30	22	24	18	32	28	0.439
BMI, mean ± SD, kg/m^2^^‡^	25.14 ± 4.17 p = 0.028	25.39 ± 4.97 p = 0.033	24.28 ± 5.13 p = 0.017	25.71 ± 4.87 p = 0.041	22.68 ± 3.25	34.79 ± 3.12 p = 0.008	28.12 ± 4.72 p = 0.029	27.33 ± 5.06 p = 0.029	28.39 ± 6.11 p = 0.021	29.12 ± 5.43 p = 0.013	0.787
Age, mean ± SD, years	25.2 ± 6.2	26.7 ± 5.5	25.8 ± 4.9	27.1 ± 4.7	26.6 ± 5.2	28.1 ± 4.7	27.7 ± 5.7	26.6 ± 6.1	27.7 ± 5.9	25.2 ± 5.1	0.916
**OGTT (75 g), mmol/L**											
Fasting level (at 8 a.m.)	6.1 ± 0.9 p = 0.009	6.4 ± 0.7 p = 0.004	6.2 ± 0.5 p = 0.003	6.1 ± 0.7 p = 0.008	3.8 ± 0.5	4.4 ± 1.2 p = 0.078	8.4 ± 0.9 p = 0.008	8.7 ± 1.1 p < 0.001	9.2 ± 0.6 p < 0.001	8.8 ± 0.8 p = 0.007	0.619
1 h	9.9 ± 1.8	10.1 ± 1.7	10.3 ± 1.4	10.5 ± 1.1	N/A	N/A	N/A	N/A	N/A	N/A	0.988
2 h	8.9 ± 0.7	8.7 ± 0.9	9.2 ± 0.6	9.4 ± 0.7	N/A	N/A	N/A	N/A	N/A	N/A	0.731
**HbA1c, mean ± SD, %**											
I trimester	4.9 ± 0.3	5.1 ± 0.7	4.7 ± 0.6	5.3 ± 0.5	3.1 ± 1.5	3.9 ± 0.9	5.6 ± 0.7	5.5 ± 0.9	5.4 ± 0.6	5.6 ± 0.4	0.541
II trimester	6.6 ± 0.9	6.8 ± 1.2	6.1 ± 0.7	6.6 ± 0.7	3.5 ± 1.4	4.1 ± 0.3	8.8 ± 0.7	8.3 ± 0.9	8.6 ± 0.6	7.9 ± 1.1	0.491
III trimester	6.3 ± 0.6	6.6 ± 0.7	5.9 ± 0.8	6.1 ± 0.4	3.9 ± 0.9	4.4 ± 0.5	6.7 ± 0.6	6.6 ± 0.4	7.2 ± 0.5	6.5 ± 0.8	0.762
Maternal weight gain, kg	9.7 ± 5	11.4 ± 6	10.8 ± 3	12.5 ± 5	10.1 ± 3	12.3 ± 3	11.8 ± 5	10.4 ± 4	13.1 ± 3	9.8 ± 5	0.681
Fetal weight, mean ± SD, g	3178 ± 230	4201 ± 206	3212 ± 109	3950 ± 330	3207 ± 112	3169 ± 250	4127 ± 287	3160 ± 230	4297 ± 231	3156 ± 179	0.322

The groups were aligned at BMI value which was measured between 16 and 19 weeks of gestational age; obesity was qualified if BMI exceeded 31 kg/m^2^. The control group (G05) was represented by patients with uncomplicated pregnancy course and did not exceeded fasting glucose level of 5.8 mmol/L and gave birth healthy newborns with no signs of diabetic fetopathy or other perinatal pathologies observed during pregnancy or postpartum. The 75 g OGTT was conducted according to the recommendations of IADPSG (revision 2010) adopted by Russian Association of Obstetrician and Gynecologist (revision 2012). Antenatal complication in form of diabetic fetopathy was diagnosed by ultrasound examination between 22 and 35 weeks of gestational age and confirmed in postpartum using Apgar-1 and Apgar-5 score test.

^¶^In obese group (G06) only patients with uncomplicated pregnancy and normal outcome were included for the study.

^†^Details for diagnostic criteria of diabetic fetopathy established in patients with GDM, T1DM and T2DM are given in Material and Methods section; *NC* normal course of gestation; *DF* diabetic fetopathy.

^‡^The indicated BMI was measured between 16 and 19 weeks of gestational age.

^★^iGDM—patients with GDM managed by insulin therapy.

^∇^dGDM—patients with GDM managed by dietary intervention∇.

Totally, we have identified 175 plasma proteins that were shared between all studied groups. The majority of them are typical plasma proteins taking the roles in transport of different ligands, signal transduction, extracellular matrix constitution, and mediating the immune response. In an overwhelming majority, biological systems are represented by a complex array with multiple co-influencing and interplaying effects that cannot always be determined precisely or numerically. We used descriptive statistics on the pilot steps to perceive samples under consideration to an aligned condition with a probability density distribution close to the normal (Appendix A). Dozens of proteins demonstrated a meaningful statistical difference between the studied groups (Fig. 1) and were accounted for the semi-quantitative analysis with the significance cut-off of p < 0.01.

The measured ranges of protein abundances can be wide enough and may cover up to one order of magnitude that causes the need for additional corrective actions in statistical analysis. The error assessments for each protein of the study groups varied somewhat depending on the group of study. Some subjects within every group were excluded as outliers based on the baseline's significant bias (Appendix A). Among them, *SERPINA1, A2M,* and *AGT* fluctuations are known to be associated with embryogenesis and pregnancy condition and long-term diabetes mellitus.

### Intragroup correlation and exclusion of outliers

Misrepresentations in the introductory statistics can be caused by many reasons: hidden pathophysiologic processes, subclinical infections, and peculiarities of the gestation course. Therefore, patients within each separate group (Table 1) were analyzed by the rank correlation analysis to evaluate the convergence of distribution within a particular group and to reveal the discriminating signs between examined groups since, as expected, patients within individual groups should have a similar distribution of features. Complete protein distribution across study groups is provided in Appendix A.

Some groups (G03M, G04M, G01P) demonstrated the correlation between subjects from 0.29 to 0.33 and 0.41. In the G04M group, only one patient (number 80) turned out to be the least relevant, and the minimal correlation was presented with patients 50 and 06 (r^2^ = 0.69 and r^2^ = 0.67, respectively). Group G01P was attributed by seldom correlation reached in a pair of patients 69 and 26 (r^2^ = 0.59). Some patients in groups G02M, G07M, G03P, G04P were excluded from the further consideration because of the increased relative abundance of specific proteins, among which the most striking were *PON1*, *CFH*, *HRG*, *JCHAIN*, *A1BG*, *TF*, and *IGKV3-20*. The pattern of these proteins in the excluded patients was approximately twice exceeding the mean pattern. Eventually, after the correlation analysis, the assayed groups were treated for systematization and alignment based on the distribution of protein abundances.

We observed that the typical reason for intragroup biases of correlation was caused by a reduced concentration of various proteins: *F2*, *CFB*, *CP*, *IGHM*, *APOC3*, and *IGKC*. Since these proteins are represented in LDL and HDL transport, copper transfer, and acute, it could be suggested that such a patient has a chronic hepatopathy. However, the relative abundances of *A2M* and haptoglobin were only slightly out of range: 0.386 vice 0.431 and 0.951 vice 1.135 in relative units, respectively. On the other hand, a decrease in the relative abundance of transport proteins and immune proteins might indicate a systemic disorder related to lipid and electrolyte metabolism, leading to nephropathy and autoimmune response. It was assumed other associated diseases that had a rather strong influence on each irrelevant patient's proteomic profile and, accordingly, the constitutive profile of the study groups.

### Intergroup correlation

The intergroup correlation by Kendal's *tau*-analysis allows comparing all groups of both series within a compiled symmetric matrix. It was necessary to elect commonly identified proteins that constitute dynamic proteome across the study groups within each examined series. The resulting proteome patterns were made of 30 proteins in the M-series and 77 proteins in the C-series (Appendix B). The average distance between patients within the same study group of M-series was d^2^ = 0.189. In the C-series, the average distance between patients within the same study group was d^2^ = 0.204, reflecting the effectiveness of the previous correction actions.

In the M-series, the maximum correlation was observed in the cluster of G01M-G04M groups, where Kendal’s coefficient was t^2^ = 0.80–0.86, and between G02M and G09M (t^2^ = 0.94) (Fig. 1). Group G09M was significantly varying by minimal correlation with control G05M-G07M, where the correlation was negative (t^2^ = − 0.10) (Fig. 1). The obesity group (G06M) was minimally correlated with all other studied groups (Appendix B).

In the C-series, group G03P was the most explicit example of a negative correlation toward G02P, G04P, G06P-G10P (Fig. 1). The maximum Kendal's correlation cluster reached between groups G04P, G06P, G07P, G09P (t^2^ = 0.89–0.64) while the strongest negative correlation was reached between G06P, G07P, G02P, G04P (t^2^ = − (0.56–0.40)). Complete correlation data are shown in Appendix B.

### Construction of the dynamic proteome heatmaps

Details of dynamic proteomes evoke an increasing interest for further consideration. Therefore, data structured after the rank correlation analysis and corrective alignment used for quantitative estimation of protein abundances accounted as fold changes compared to the control group (G05). The Gene Ontology analysis in support of KEGG pathways mapping was carried out to determine proteins network of interactions utilizing their molecular functions and biological processes interconnected with metabolic pathways in which specific proteins are involved.

**Figure 2:**
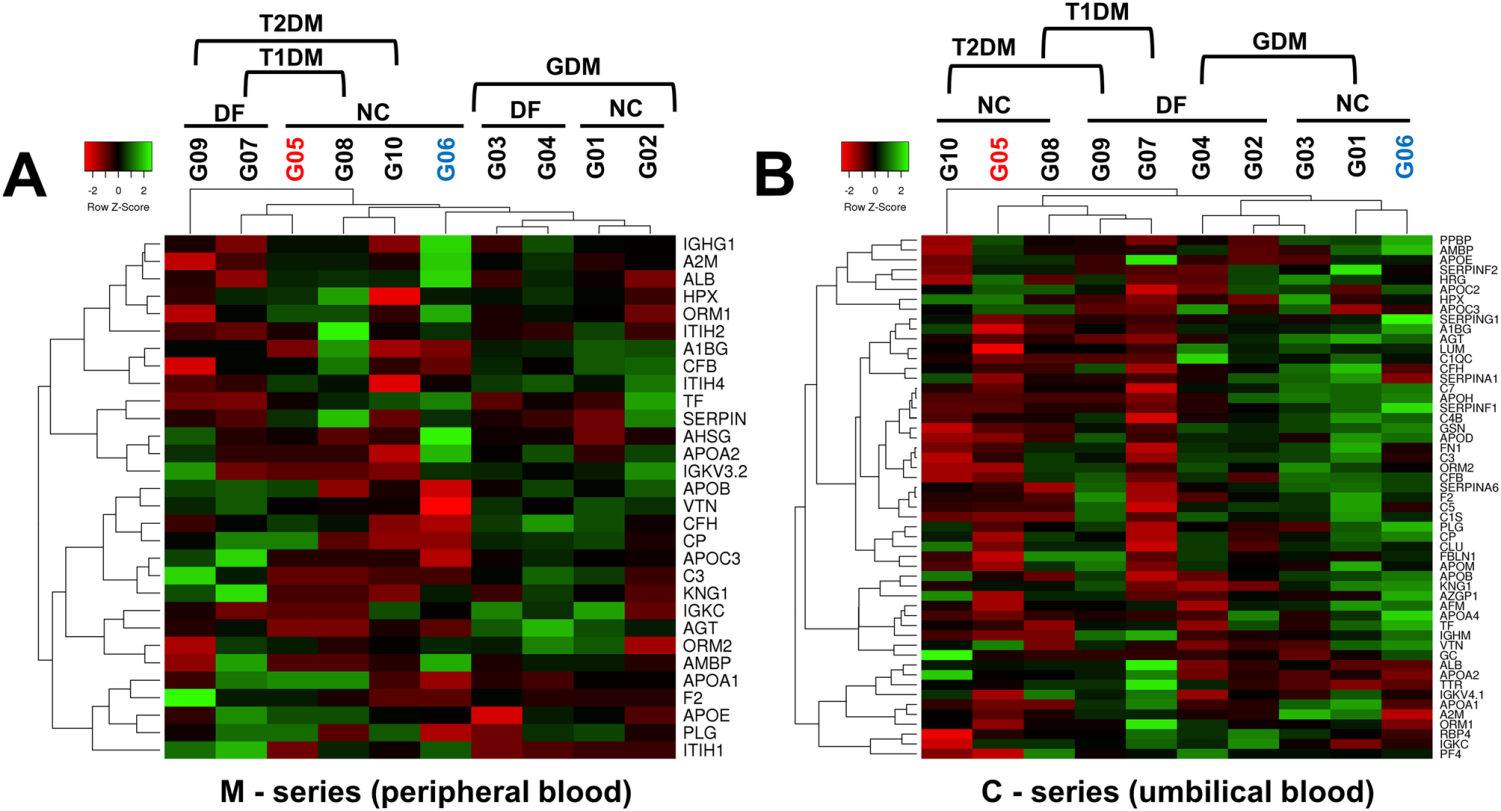
Heatmap of the differentially altered proteome implicated in the initiation and progression of the diabetic condition during gestation and its impact on dire antenatal consequences. **(A)** In the M-series (peripheral blood samples) of study groups, the differential semi-quantitative map includes proteins that differ between groups from 2.5 to 0.5-fold changes (at p < 0.005). The hierarchical clustering demonstrates the closest relation between groups of GDM (G01M-G04P) based on the dynamic changes of the most abundant proteins. Groups of T2DM (G07M and G09M) are the most detached due to the contrast dynamic of proteins involved in hemostasis maintenance and the complement cascade. The sparsely integrated group is G06M (obesity), demonstrating a specific increase of major plasma transport proteins. **(B)** The C-series (umbilical blood samples) study groups the presented differential semi-quantitative map includes proteins that differ between groups from 2.5 to 0.5-fold changes (at p < 0.005) while a portion consisted of 22 proteins with p < 0.01 was excluded from this map. The hierarchical clustering demonstrates that the control group (G05P) appears to be the most detached group characterized by a significant decrease of the vast fraction of proteins under consideration. At the same time, some groups of GDM (G01-G03P) are closely related to T2DM patients who delivered newborns with signs of DF (G09P) by similar dynamic among proteins involved in the lipid transport and inflammatory condition. In contrast, other GDM group (G04P) was outlined and behaved in a counter wise fashion to the obesity group (G06P) and T2DM patients with the normal outcome of pregnancy (G10P).

Heatmaps of the dynamic distribution of the relative protein abundance across study groups within the M-series and the C-series are demonstrated in Fig. 2. Having tracked the dynamic changes across the M-series (Fig. 2A), we focused on a cohort of *CP*, *PLG*, *SERPINA1*, *KNG1*, *AGT*, *PLG*, *APOE*, *ORM1*, *TF*, and *VTN*. These proteins exhibited tremendous variability through all groups of M-series. In the C-series (Fig. 2B), the most varying proteins were *APOM*,* CP*, *PLG*, *AGT*, *KNG1*, *APOA1*, *ORM2*, *TF*, *HRG*, *APOD*, and *LUM*.

The majority of proteins defined in the M-series were characterized by peptidase, hydrolase, and catalytic activities, and most of them are involved in response to external stimuli (Appendix C). In the C-series, the attitude was more complicated and included proteins involved in the signal transduction and receptor binding, whereas proteins responsible for the antigen-binding activity were explicitly emphasized. Both the series were featured in proteins of nitrogen metabolism and receptor-mediated immune response (Appendix C). Proteins involved in signal transduction through cell surface receptors, including Fc-mediated, are also meaningfully distinguished.

### Differential semi-quantitative analysis of markers in the peripheral blood samples

A little more than 20 different protein identifications were selected as potential markers associated with diabetes mellitus and the possible risk of DF. Data in Table 2 and Fig. 2A demonstrate the relative abundance alterations (in FC, fold changes) of proteins selected from the M-series groups at a confidence level of p < 0.01 (unless other is indicated in Table 2).

**Table 2:**
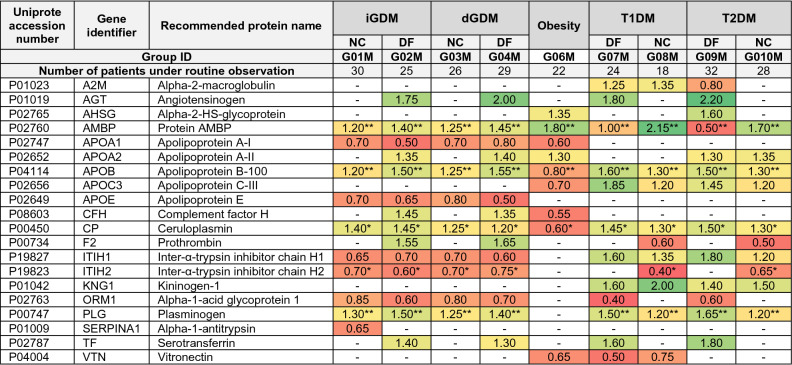
Alterations (in fold changes, FC) of proteins relative abundances in groups of the M-series (peripheral blood samples).

The FC are normalized to the control group (G05M, uncomplicated pregnancy, normal outcome) and the data are represented at p < 0.01 unless other is specified in the table’s footnote. Patient with GDM were subdivided according medication strategy: insulin therapy (iGDM) or dietary intervention (dGDM). Apart obesity group (G06), all other studied groups were also stratified according pregnancy outcome in regard of the presence of diabetic fetopathy (DF) or absence (normal course) the signs of DF and other antenatal pathologies observed during pregnancy and in postpartum.

*p < 0.005; **p < 0.001.

#### Alterations in lipids transport and signal transduction proteins

The immediate attention is turned to the apolipoprotein family comprised of *APOA1*, *APOE*, *APOA2*, and *APOC3*. The abundance of *APOA2* was 30–35% higher in groups with T2DM (G09M and G10M) than in the control group. Besides, there was no significant change in groups with T1DM (G07M and G08M), assuming the association of *APOA2* with T2DM. Nevertheless, one cannot ignore that groups G02M and G04M (GDM accompanied by DF) demonstrated an increase of *APOA2* of 20–25% (p = 0.00079). An increase of up to 30% also was indicated in group G06M (obesity). It should be emphasized that the most prominent increase was detected in groups attributed to DF (Table 2).

Although *APOA2* and *APOC3* are members of the PPAR signaling pathway, they showed utterly different behavior. Unlike *APOA2*, the abundance of *APOC3* increased by up to 45% in the group G09M and up to 85% in the group G07M. Both groups are characterized by newborns with DF but enroll patients with different types of diabetes mellitus. The lower range was indicated in newborns with no signs of neonatal pathologies (G08M and G10M), whereas groups of GDM positive patients (G01M–G04M) did not demonstrate significant alterations in *APOC3* level. The only decrease of 30% was observed in group G06M.

#### Alterations in proteins of the pro-inflammatory condition and cholesterol clearance

The most prominent dynamic was designated for regulatory protein *AGT* but only in groups with GDM (G02M, G04M) and in the group with T1DM or T2DM (G07M and G09M) associated with DF (Table 2 and Fig. 2A) where the increase was in a range of 1.50–2.00 folds change (p = 1.37e−04). Aside from group G06M, the *CP* increased by 20–40% throughout all study groups. Having mentioned the *CP*, we have to fairly note *TF*, which increased by 30% but specifically only in groups with GDM with healthy newborns (G01M and G03M, Table 2).

The pronounced alterations were demonstrated in the relation of *APOA1* and *APOE*. Both proteins participate in PPAR-mediated signaling and decreased by 30–50% only in groups with GDM (G01M–G04M) and did not demonstrate a significant level change in groups with T1DM and T2DM. The activity of protein *APOE* depends on *A2M* (Table 2), of which abundance was reduced by 20% only in the G09M group (T2DM with DF consequence) and meaningfully elevated in T1DM patients (G07M and G08M) irrespective of antenatal complication. An increased level of *APOB* was indicated in all tested groups (Table 2). Interestingly, an increase of up to 60% was observed in the groups with DF (G02M, G04M, G07M and G09M), whereas patients with a normal fetus development (G01M, G03M, G08M, and G10M) depicted an increase of 30% at maximum.

#### Influence on the hemostasis regulation

There were several biomarkers represented in the regulation of hemostasis (Table 2 and Fig. 2A). In this study, the most articulating changes were found in T1DM and T2DM groups (G07M, G08, G09M, and G10M), whereas groups of GDM (G01M–G04M) exhibited mild changes as for *PLG* and *F2*, or lack of changes as for *KNG1* (Table 2). The *PLG* was emphasized by elevation specifically in groups with signs of DF: up to 40–45% in the groups G02M and G04M, up to 50% in the group with T1DM (G07M), and up to 65% in T2DM (G09M). The most striking changes that unite a significant increase of all hemostasis-related proteins (*PLG*, *F2*, and *AGT*) were populated in patients with T2DM and signs of DF (G09M, Table 2).

Alterations of *F2* and *AMBP* perfectly matched since both demonstrated similar dynamic changes. The *AMBP* increased up to 40% in all groups with GDM (G01M-G04M), and the most meaningful alterations were detected in groups of T1DM and T2DM patients that delivered healthy newborns (G08M and G10M). The opposite situation was demonstrated if we consider *F2*, which elevated in GDM patients who delivered DF newborns (G02M and G04M), but decreased levels in T1DM and T2DM patients with healthy newborns (G08M and G10M, Table 2). The analysis of the co-expression pattern in the STRING revealed that the closest co-expressing neighbors of *AMBP* are *APOC*3 (0.863), *APOA2* (0.852), and *APOB* (0.696).

### Differential semi-quantitative analysis of markers in the cord blood samples

Only 41 proteins were retrieved for further consideration from the exhausted proteome of C-series. The widely represented family of apolipoproteins in the C-series was significantly extended (Table 3 and Fig. 2B). Some apolipoproteins draw attention to being unique representatives in the C-series: *APOD*, *APOM*, *APOA4*, *APOC2*. The first two proteins are the least scrutinized, and information about their functional role is limited and ambiguous in the literature (Table 3).

**Table 3:**
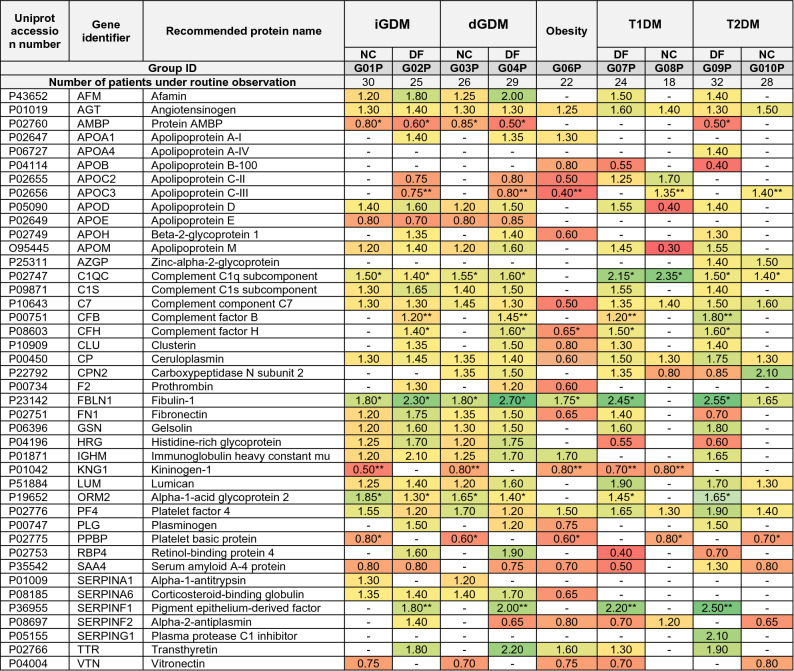
Alterations (fold changes, FC) of proteins relative abundances in groups of the C-series (umbilical cord blood samples).

The FC are normalized to the control group (G05P, uncomplicated pregnancy, normal outcome) and the data are represented at p < 0.01 unless other is specified in table footnotes. Cord blood samples were stratified according the clinical finding of manifested types of diabetes mellitus in donors and signs of diabetic fetopathy (DF) or absence of DF and other observed antenatal pathologies. Samples were not stratified according the way of birth delivery since no significant correlation was observed between the way of delivery and DF frequency or type of diabetes mellitus, however mean ratio of Cesarean birth to natural delivery of newborns was consisted 59.4% (considered a sum of planned and emergency delivery).

*p < 0.005; **p < 0.001.

#### The impact on transport of 9-*cis*-retinoic and bile acids

The duet of *APOM* and *APOD* rendered the most prominent intergroup alterations. Both proteins were overrepresented in all groups, aside from the group of obesity (G06P). In groups of GDM with DF complication (G02P and G04P) abundance of *APOM* increased up to 40–60%, and up to 50–60% for *APOD*, whereas GDM groups with healthy newborns (G01P and G03P) revealed up to 20–30% affordable increase of both the proteins. An increase within a range of 30–40% was observed in groups with T1DM and T2DM with the manifested complication of fetal growth and development (G07P and G09P, Table 3). Additionally, both *APOC2* and *APOC3* diminished by 20–30% in groups of GDM patients who gave birth to newborns with DF signs (G02P and G04P). It should be noted that unlike M-series, *APOC3* recurred increased alterations not only in the G06P group (obesity) but a decrease of at least 30% was also noted in patients with GDM and signs of DF (G02P and G04P, Table 3).

#### Stimulation of extracellular architecture remodeling and morphogenesis

The extracellular protein *FBLN1* reflected increased background in GDM groups by 80% (G01P and G03P) and even by 120–170% (G02P and G04P) in case of manifested DF. Other groups with DF and related to either T1DM or T2DM (G07P and G09P) were demonstrated the same tendency and made up to 155% increasing level change. Meanwhile, *APO*H increased up to 40% in groups G02P, G04P, and in the group of patients with T2DM who gave birth to newborns with signs of DF. Oppositely, the *APOH* decreased by 40% only in group G06P (obesity).

The neurotrophic regulator of Wnt-signaling, *SERPINF1*, demonstrates substantially increasing concentration in all groups where examined patients delivered newborns with antenatal complications and reached up to twofold changes in GDM groups (G02P and G04P) and up to 2.5-fold changes in T1DM (G07P) and T2DM (G09P) patients. Nearly the same behavior can be traced for the *TTR*, which is elevated only in groups with signs of DF but displays more prominent alterations in GDM patients (G02P and G04P). In contrast, patients with T1DM (G07P) and T2DM (G09P) demonstrate less extensive changes in its concentration (Table 3).

#### Activation of cytokines and pro-inflammatory condition

The abundance of *CP* increased by 30–50% in groups with GDM (G01P–G04P) and by 50–75% in the group with T1DM or T2DM designated by DF newborns (G07P–G10P). However, in the obesity group (G06P) significant change was not detected, despite explicit alterations in the M-series (Table 3, Fig. 2B). The protein *AGT* was enlarged by 30–40% in groups with GDM (G01P–G04P) and with T1DM and T2DM (G07P and G09P) precisely as in M-series. However, there was no significant difference between patients with healthy or affected by DF newborns. The conduct of *HRG* in the dynamic proteome largely repeated the trace of *AFM*: both were characterized by a significant upward (70–75%) in the GDM groups with DF signed newborns (G02P and G04P) whereas relative abundance of HRG significantly reduced below 40–50% in T1DM and T2DM groups with DF (G07P and G09P), and almost unaltered in groups with healthy newborns (Table 3).

The *C7* complement factor increased in all study groups (except the G06P) by 30–50%. However, other members of complement system regulation, *CFH* and* CFB*, displayed identical alteration and increased only in the group of patients with the affected newborns (G02P, G04P, G07P, and G09P). Contemporaneous elevation levels of *PLG*, *F2* and *IGHM* up to 20–50% was exhibited specifically in G02P and G04P Table 3) whereas patients with T1DM were distinguished by unaltered abundancies of these proteins. The G09P differed by a higher (up to 50–60%) abundance of *PLG* and *IGHM* compare to the GDM patients but unchanged constraints of *F2*. The G06P (obesity) group exceptionally displayed a decrease of 70–75% of both *PLG* and *F2*.

#### Alterations in bloodstream transport proteins

The gap between immune response and immunoglobulins clearance was filled by *ORM2*, of which the observed relative abundance was characterized by differential up-regulation in groups with GDM: 60–70% (G01P and G03P) and up to 40% (G02P and G04P). Groups with T2DM did not display deviations from the control group G05P. Increased abundance (up to 40–45%) of *SERPINA6* was discovered in all groups with GDM (G01P-G04P), and to less extent (up to 30%) in patients of T2DM with DF newborns (G09P). In the obesity group G06P, a decrease of 35% also noted.

## Discussion

### Proteomic map of peripheral blood samples

Apparently, that customized elements of proteome populated from the M-series samples make it possible to segregate groups with GDM from those with T1DM and T2DM and may support assessing the potential risk of DF complication. The GO analysis splits elements of the M-series proteome into several major biological process among which coagulation (FDR = 7.02e-11) and metabolism of lipids and their derivatives (FDR = 4.33e-12) were the most represented following negative regulation of hydrolysis activity (FDR = 1.52e−10), regulation of plasma lipoproteins (FDR = 3.01e-08) and level of lipoprotein particles (FDR = 3.71e-07). According to molecular functions stratification, the most extensively represented activities were endopeptidase inhibition (FDR = 2.29e−09), binding to receptors (FDR = 3.53E−07), and cholesterol carriers (FDR = 0.00014). The majority of proteins are typically localized in the extracellular space (including secretion with exosomes), and some are being residents for plasma (Appendix C).

Diabetes mellitus is a highly complex pathology that generally encompasses disturbance of lipid and carbohydrate metabolic processes and impacts immune response modulation, leading to chronic inflammation. Thus, we aimed to combine the detected proteins into a single chain of molecular events and trace this chain together with the estimated quantitative alterations and the attributed biological processes.

#### PPAR-mediated clearance of lipids and glucose exchange

**Figure 3:**
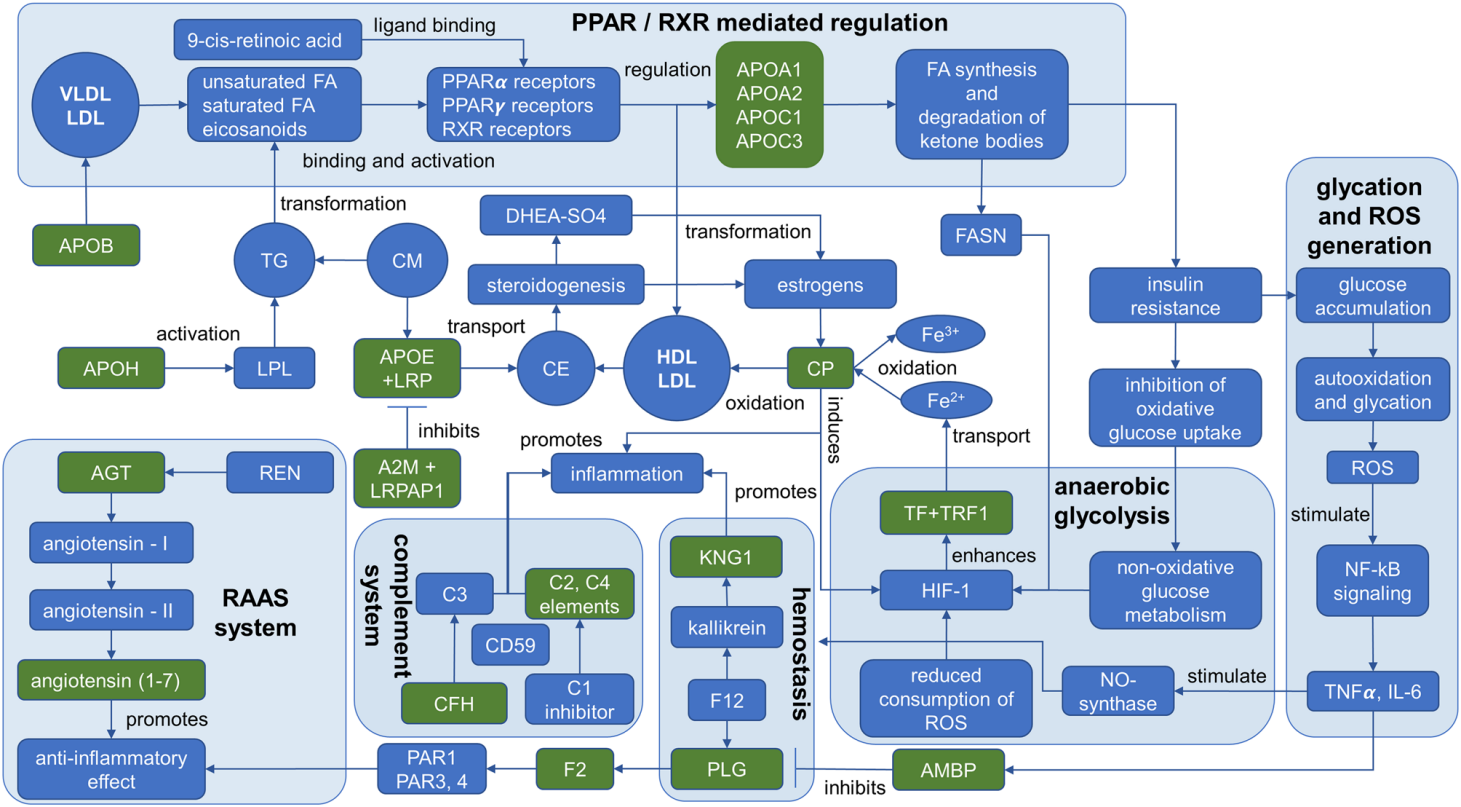
Reconstruction of the proteomic map of peripheral blood that reflects the molecular processes commonly involved in the progression of GDM, T1DM, and T2DM and accompanying the complication of fetal development. The impaired glucose uptake and consequent insulin resistance is mostly affected by disturbance of lipid transport and faulted lipid metabolism in which apolipoproteins play a crucial role. In diabetic condition, glucose utilization is shifted to non-oxidative glycolysis which is rendered in the enhanced lactate and pyruvate interconversion leading to tissue and endothelium damage, and to de novo lipids synthesis due to the corrupted insulin clearance. This can be detected in the imbalance of VLDL, HDL, and LDL maintenance and progression of inflammatory-related processes enhanced by the oxidative properties of *CP*. The level of *CP* may also increase due to the gained production of estrogens during gestation. The developing insulin resistance creates a risk of blood glucose accumulation, its autooxidation, protein glycation, and oxidative stress. This leads to further deployment of the pro-inflammatory condition in the way of HIF-1 induced activation of *TF* through *TRF1* receptors up to the late antenatal age. The positive regulation loop is accomplished through the enhanced synthesis of estrogens, which upregulate *CP*. The effect of consequent tissue damage and inflammation due to oxidative stress caused, partially, by glucose autooxidation is enhanced by the coupled activation of *KNG1* and *PLG* positively regulated by *AGT*. On the other hand, the enhanced expression of *AGT1* brings to an anti-inflammatory effect through functional interaction with F2. In general, these interactions place the hemostasis at a higher risk of thrombosis, which is also enhanced by the action of AMBP inhibiting PLG. Positive feedback is accomplished through the increased *APOH*, which activates the LPL powering generation of fatty acdis (FA) from triglycerides (TG). In turn, the latter closes the loop with PPAR receptors guiding the lipid metabolism and glucose uptake.

Interactome analysis demonstrated a direct relationship between *APOA1, APOE, APOA2,* and *APOC3* in an attitude of clearance of lipoprotein particles and steroid synthesis (p = 5.76e−08). In this context, not surprisingly, that apolipoproteins family was the most prevalent and disclosed the most valuable features (Table 2 and Fig. 2B). Apart from *APOE*, all other mentioned apolipoproteins are involved in the clearance of circulating lipids by regulating gene expression through PPAR-signaling (Fig. 3). PPAR are nuclear receptors regulated by fatty acids or their derivatives^15^ and exist as three functional groups: α-type is involved in the clearance of circulating lipids by regulating gene expression in the liver; β-type are involved in lipid oxidation and cell proliferation; γ-type provide differentiation and activation of adipocytes to increase the exchange of blood glucose. Earlier, proteins of the PPAR-pathway were encouraged with growing interest because the increasing expression of PPAR-related mediators was associated with diabetes mellitus^16,17^ and with GDM in clinical practice (p < 0.001), albeit data were based on the assay for omental visceral adipose tissue^18^. The level of *APOA2* and *APOC3* is affected by PPAR-mediated signaling^16,18^. We found that the level of *APOA2* was non-specifically increased in all studied groups, including the groups of obese patients (G06M). However, specific alterations were observed in case of GDM positive groups (G02M and G04M, Table 2). Therefore, we supposed that *APOA2* can be associated with DF signs if increasing level was detected in GDM positive patients. In contrast, *APOC3* was instead noted in patients with T1DM and T2DM and displayed a risk of DF if its level indicated above the baseline compare to the control group (Table 2).

In turn, protein *APOA1* is directly implicated in lipids degradation and oxidation, whereas *APOE* plays a crucial role in the catabolism of IDL, LDL, and VLDL, and plasma-tissue lipid transport (Fig. 3). PPAR-receptors induce the *APOE* expression in coordination with RXR-receptors activation in most neurons and liver tissue^19^. Both proteins influence glucose and lipid metabolism and impact the development of type III hyperlipoproteinemia, vascular dementia, and ischemic stroke^20^. There is no strong association of *APOE* with consequences caused by T1DM. Still, there is evidence for an elevated risk of vascular dementia in patients with T2DM^20,21^ due to the rising concentration of *APOE*.

The results of our study somewhat contradict those reported before^20,21^ because we detected diminished abundances of *APOE* in GDM positive patients (Table 2) and unaltered levels in T1DM and T2DM patients. Nevertheless, despite unequal molecular functions of *APOE* and *APOA1*, both can be flagged as potential markers of GDM, while DF progression can be supported by the increasing of *APOA2* (Table 2).

#### Complement factors and concomitant RAAS activity promote insulin resistance

Pregnancy is accompanied by multiple re-arrangement in endocrine signaling, immune system, and impacts on the coagulation system. It is known that the renin–angiotensin–aldosterone system (RAAS) and its regulator *AGT* are stimulated during pregnancy. Recently, the results of transcriptional analysis^10,22^ showed an increased expression of genes associated with the maturation of angiotensin (p < 0.0005) in T1DM patients^23^. However, it was demonstrated^23^ the suppression of angiotensin-(1–7) and lowering the ratio to angiotensin-I in GDM patients cause endothelial dysfunction during gestation and in the postpartum period. Our study revealed the distribution of *AGT* specifically among GDM groups associated with DF (Table 2) while the highest level was indicated for T2DM patients (group G09M, Table2). This observation agrees with the finding that the risk of T2DM onset following GDM can be effectively prevented by ACE inhibitors and angiotensin-receptor blockers^24^, suggesting the excessive *AGT* in the course of GDM. Simultaneously, the combination of *AGT* with the *APOB* increased by more than 50% can foster to account *AGT* as an indicator of DF in all studied groups irrespective of the type of diabetes mellitus (Table 2). There is no direct relation between *APOB* and *AGT*, but it is known that *APOB* binds with the cell surface receptor SORT, which is also a co-receptor for SAPS^25,26^. Both proteins concurrently bind with the same receptor to accomplish endocytosis, but they are functionally unrelated. If *APOB* mediates transport of LDL and cholesterol clearance, the role of SAPS mainly involved in GRN-mediated exocytosis of lysosomal and peroxisomal proteins^26^. Moreover, the disturbance of such exocytosis mechanism induces a boosted reaction of cytokine activation and, consequently, the development of pro-inflammatory conditions through the activation of the complement system^27^.

Thus, there are at least two proteins (*APOB* and *AGT*) that impact the activity of the complement system in our study; however, angiotensin regulates the stability and inhibition of the complement system (Fig. 3, Appendix C)^28^. In turn, experimental reports support the interconnection between the complement system and their contribution to the dysregulation of adipose tissue metabolism^29,30^. The abundance of *C3, C2*, and *C4* factors due to glycation-mediated inactivation of *CD59* is the most extensively known mechanism of complement-mediated tissue damage in T1DM and T2DM patients^31,32^. It is known that patients with T1DM or T2DM are characterized by a decreased level of C1-inhibitor^33^, and increased *C2* and *C4* fractions^34^. Association between increased expression of *CFH*^34^ in adipose tissue with insulin resistance (IR) in T2DM patients has also been recently reported^35,36^. Subjects with induced IR and HOMA-IR values larger than 3.9 were characterized by overexpression of *CFH* to 0.59 ± 0.12 against a baseline of 0.35 ± 0.16 (p = 0.003)^36^. This study found that that *CFH* became prevalent specifically in groups of GDM patients with the affected newborns (G02M and G04M; Table 2, Fig. 3). Thus, it can be assumed that *CFH* can be attributed to IR and the possible risk of DF in GDM progression.

In summary, we suggested that the alternation in levels of complement system elements can be caused by both the increased stoichiometry ratio of *APOB* that inclines positive regulation of cytokines secretion and by regulation of the immune response through *AGT*^25^ (Fig. 3). Our findings suggest implication of *APOB* and *AGT* in the progress of hemostasis impairments and the supression of immune response, specifically indicating the risk of DF. However, complement factors are ubiquitously detected in proteomic experiments since they belong to the fraction of abundant plasma proteins. Therefore, we cannot suppose confidently whether the observed factors were directly linked with the GDM condition or caused by other cryptic factors.

#### Escalation of oxidative stress and inflammatory condition

Despite the confident detection of *CP*, it is not easy to consider for this protein solely as an evident and robust marker of the pathogenesis of diabetes mellitus, since many factors can stress its increase. Estrogens stimulate synthesis of *CP*, so its abundance may elevate in the course of gestation^37^. Depending on the circumstances, *CP* can act as a prooxidant or antioxidant. In the presence of reactive oxygen species, the protein acts as a catalyst for LDL oxidation^38^. Besides, *CP* is a mediator for generating oxidized Fe^3+^ essential for lipids oxidation via Fenton's reaction^39^. This may lead to substantially boosted production of reactive oxygen species stimulating NO-synthase and damaging tissues and vessels^39,40^ (Fig. 3). While progressing GDM, an adverse impact in iron and nitrogen balances can happen. Severed impairment of iron metabolism enhances *CP* expression via HIF-1-mediated activation of *TF* and its co-receptors^41^. Therefore, not surprising that patients related to groups with DF were characterized by the increased levels of *TF* in our research (Table 2). At the same time, HIF-1 triggers activation of non-oxidative (anaerobic) glycolysis which is typically significantly enhanced in patients with T2DM and GDM and causes lowering the acid–base balance due to excessive generation of lactate as an end-product of glycolysis^42^.

Generally, these events arrange the condition for the further progression of pro-inflammatory reaction through the escalation of the complement cascade and enhanced by dysregulation of RAAS. We supposed that compensation of the depleted *CP* is a necessary condition to prevent deployment of oxidative stress during gestation while the elevated *TF* may indicate the risk of DF. Hence, if depletion of *CP* would not be sufficiently compensated, it may follow to the escalation of inflammation through NF-kB signaling and to the dire consequences in fetal growth and development^43,44^. Therefore, as expected, we observed a significantly elevated *TF* level as an alarming feature in patients with the affected newborns (G02M, G04M, G07M, and G09M, Table 2).

#### Stressed hemostasis

Insofar patients with diabetes mellitus are characterized by the affected hemostasis^45^, this pathology is qualified as a prothrombotic condition with a high risk of cardiovascular diseases. Stressed hemostasis is primarily coupled with an impact on the faulted carbohydrate metabolism^45,46^ and rising oxidative stress caused by blood glucose autooxidation and, consequently, proteins glycation^44^. Such patients demonstrate the growing prothrombin, even though alterations in activity and abundance of *KNG1* are seldom observed^46^. The clinical finding may be aggravated by ketoacidosis, which brings thrombosis and damages endothelium due to the activation of the coagulation system (Fig. 3). Thereof, proteins involved in hemostasis balance typically exhibit overrepresentation in T1DM and T2DM groups in contrast to GDM^47,48^. However, turning to the panoply of the obtained data, the lack or moderate changes in *F2* and *KNG1* abundances can be considered as a low-risk indicator of DF throughout the study groups (Table 3). Simultaneously, if *PLG* increased solely, moderate risk of DF can be suspected; and high risk assumed if elevated levels for complete cohort (*F2*, *KNG1* and *PLG*) of proteins were observed (Table 2).

Extended analysis of the reviewed proteins disclosed the expected co-expression match between *F2* and *AMBP* (co-expression 0.87). The latter is of great importance for monitoring pregnancy since its concentration gradually declines with an increase in fetus size and reaches a minimum at the end of gestation^49^. The increased concentration of *AMBP* is generally considered an indicator of pre-eclampsia and reported for patients with diabetes mellitus which agrees with the recorded rising abundance of *AMBP* (Table 2). Due to plasmin inhibition activity, *AMBP* enhances the risk of thrombosis, disturbances in hemostasis and, finally, reveals the pro-inflammatory properties^50^. Therefore, the co-occurrence of *F2* and *AMBP* was occasionally the most explicit GDM positive patients who gave newborns with signs of DF, although a rising abundance of *AMBP* was detected in all groups under consideration (Table 2).

### Proteomic map of umbilical blood samples

#### APOD- and APOM-mediated defense mechanisms through RXR/PPAR-receptors

**Figure 4:**
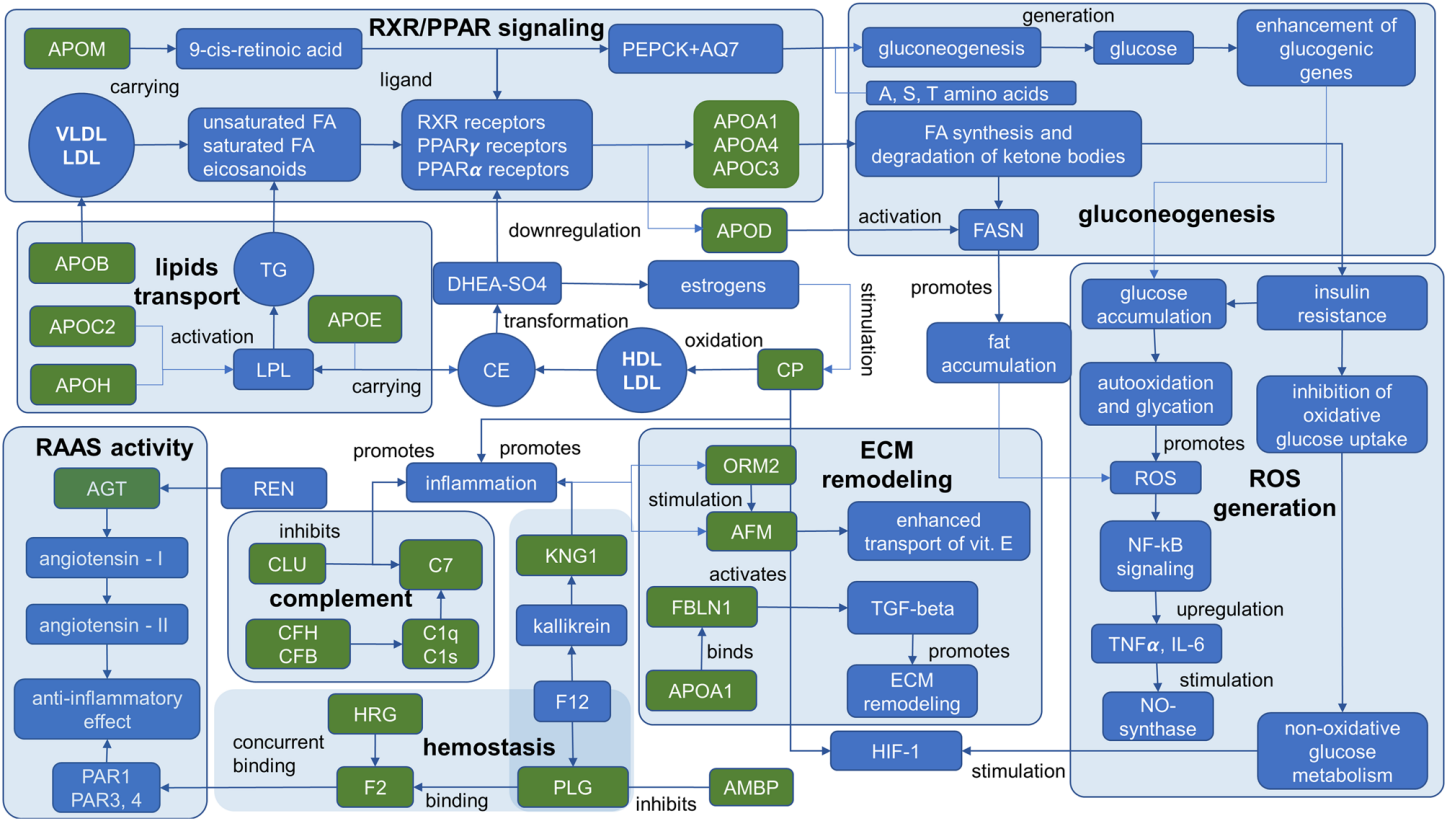
Reconstructed proteomic map in umbilical blood for molecular processes putatively involved in the complicated fetal growth and development in patients with signs of T1DM, T2DM, and GDM. The metabolism of lipids is regulated by ligands of PPAR-α receptors targeting expression of apolipoproteins. In turn, FA through activation of PPAR-γ receptors and 9-*cis*-retinoic acid transported by *APOM* to RXR-receptors may regulate expression of *PEPCK* and *AQ7* genes involved in gaining the gluconeogenesis and, consequently, enhance expression of glycogenic genes. Unlike the T2DM patients, when the increasing anaerobic glycolysis is prevalent, in T1DM patients, the origination of oxidative stress is partially evoked by the increased oxidative glycolysis rate. Both processes disbalance lipids metabolism and call insulin resistance in different ways. Impairments of lipids metabolism are also evinced in the growth of *AFM* abundance that transports vitamin E and lipophilic molecules under conditions of deficient lipids transport. Besides, *AFM* is positively regulated by *ORM2*, which is responded to the progression of inflammation conditions caused by the CP-enhanced oxidative stress and supported by the concomitant processes in the coagulation system. In hemostasis, *KNG1* is increased, and concurrent action of *PLG* and *HRG* for binding with fibrin significantly retards fibrinolysis and enhances the risk of thrombi formation. Malfunction of hemostasis is also reflected in re-arrangements of the extracellular matrix which are accomplished through the functional complex *FBLN1* and *APOA1* mediating the activation of TGF-β signaling. Although estrogens negatively regulate *APOD*, its abundance is increased due to DHEA action on the PPAR receptors as a defense mechanism in response to oxidative stress supported by *CP* and complement system escalation.

Cord blood is a highly demanding substance for research purposes but the estimation for many proteins can be either undefined, misinterpreted, or have a wide range. As with the peripheral blood samples, several apolipoproteins were attended in the C-series (cord blood) and two of them were the most interesting for further consideration (Appendix C). Among them, *APOD* is a lipocalin typically found in macromolecular complexes with lecithin-cholesterol acyltransferase and involved in transporting of various other ligands depending on the biological context (Fig. 4). Active role of *APOD* is reduced to regulation of HDL/VHDL (60–65%) metabolism, and only in trace amounts, it can be detected in VLDL and LDL. This protein is believed an atypical apolipoprotein since its structure and localization are different from other apolipoproteins and are characterized by a high capacity for cholesterol binding. The *APOD* concentration inversely depends on estrogens, i.e., 17β-estradiol, which is substantially increased during pregnancy^51^. Recently, *APOD* has already been considered as a predictive marker of GDM, but the data were ambiguous^52^. There were reports concerning the decrease of *APOD* in patients with GDM^52^ and decreased concentration in cord blood samples of patients with uncomplicated pregnancy or excessive gestational weight gain^53^. Contrariwise, a significant increase of placental *APOD* associated with GDM was also reported^54^, assuming that it reflects activation of defense mechanisms against oxidative stress. The latter is more relevant in an attitude of the data obtained in our research when *APOD* also increased in groups with GDM, but more prominent elevation has been recognized in groups with the affected newborns (G02P, G04P, G07P, and G09P; Table 3). Our data are also supported by the previously reported results about the direct correlation between the APOD concentration and enhanced generation of ROS and RNS^55^.

Data regarding the role of *APOM* in diabetic conditions are scarcely available. It is known that *APOM* is a member of the lipid transport system and capable of the transferring of sphingosine-1-phosphate, myristic, stearic, and palmitic acid. There is evidence that *APOM* is regulated by RXR-receptors and plays a role of carrier for *trans*-retinoic and 9-*cis*-retinoic acids which are ligands of PPAR/RXR receptors (*Fig. **4*)^56^. Destructions in such signal transduction entail defects in morphogenesis of placenta, heart, and eye and may cause embryo death^57^. Proper functioning of RXR-receptors is critical for regulating lipid metabolism, adaptive thermogenesis, and regulation of gluconeogenesis^57^. Albeit, it has been reported that there was no difference in the level of *APOM* between patients with the normal and complicated pregnancy, it becomes obvious that stoichiometric ratio of *APOM*, receptors and ligands is emergingly critical for the morphogenesis in course of prenatal period due to regulation of gluconeogenesis and lipids transport^58^.

Since our results displayed the prominent between-groups match of *APOM* and *APOD* abundances fluctuation (Table 3), it was suggested that their significant increase was associated with impairments of gluconeogenesis and excessive generation of ROS which intensifies the immune response and inflammatory condition due to oxidative damage^43,44,55^. Therefore, the most elevated *APOM* and *APOD* levels were exhibited in groups with the affected fetal growth and development (Table 3). Since the branch of the gluconeogenesis pathway regulates the expression of phosphoenolpyruvate carboxylase and aquaporin-7^56,57^, it can be assumed that monitoring of glucogenic amino acids alanine, serine, and threonine would be reasonable. Meanwhile, it seems that the assembly of *APOH*, *APOC2*, and *APOC3* (Table 3) holds a privileged position of markers of DF in patients with GDM if it is supported by the increased level of both *APOM* and *APOD*.

#### Remodeling of extracellular architecture through the increased fibulin-1

Both *APOM* and *APOD* are compounds of extracellular space; thence, among other explicitly altered proteins, the *FBLN1* should also be mentioned because it occupied a leading position in dynamic changes (Table 3). The protein holds a key role in determining cell migration, structural integrity of extracellular matrix, and mediating tissue remodeling during embryogenesis through interactions with *APOA1* and *THBS1* (thrombospondin 1)^59^. Fibulin-1 (*FBLN1*) is affected by estradiol in the same manner as *CP*^60^; therefore, its concentration increased during fetal growth. More important that *FBLN1* can regulate the activity of critical for angiogenesis mediated by TGFβ signaling in a context-dependent manner^59^.

There is scant evidence about the association of *FBLN1* with the pathogenesis of diabetes mellitus because the concentration of *FBLN1* in T2DM patients (93 ± 28 µg/mL) was found to be barely lower compared to the control group (106 ± 30 µg/mL (p = 0.005))^61,62^. Such weak differences and wide ranges can hardly be perceived to establish a confident correlation between *FBLN1* with diabetes mellitus. More significant results were obtained in the study conducted on patients with T2DM when an increase of *FBLN1* and correlation with the HbA_1C_ and natriuretic peptide has been detected^63^. The rise of *FBLN1* was 2.03 folds change, which endorses our study (2.20–2.50-fold changes, Table 3). In general, the indication of *FBLN1* can be used as a prognostic marker of the risk of cardiovascular disorders, which is important for patients with T1DM and T2DM since they are at a high risk of diabetic angiopathy. This can be assumed because *FBLN1* is capable of binding with fibrinogen, thus, maintaining hemostasis and thrombosis as well as modulation of neutrophil activity (Fig. 4)^64^. It was found to be closely overlapping with fibrinogen and acts in locations of thrombi formation by supporting platelets attachment via a bridge of fibrinogen to integrin^64,65^.

Hence, it was expected to determine the elevated level of *FBLN1* in combination with *F2* and *PLG*, especially in groups of patients with the affected newborns (G02P, G04P, G07P, and G09P, Table 3). Patients who gave birth to newborns with signs of DF demonstrated substantially increased levels of proteins involved in hemostasis and displayed a higher risk of angiopathy, thrombosis, and endothelium damage (Table 3). Therefore, we suggested that *FBLN1* can be considered as a secondary response on the disturbed hemostasis due to its binding with fibrinogen activity, and can generalize groups with a high risk of DF (G02P, G04P, G07P and G09P) regardless of the etiology of diabetes mellitus.

#### Insulin resistance stresses hemostasis and immune response

In light of the direct relationship of *FBLN*1 to the structuring of the extracellular matrix and maintaining hemostasis, we examined *F2*, *PLG*, *SERPING1*, *PPBP*, and *PF4* in more detail (Table 3). The analysis of proteins interactome and subsequent functional analysis demonstrated that majority of proteins are consolidated into a single functional net (PPI p < 1.0e−16) that can be split by criterion of biological processes into two subgroups with shared elements: the compliment (FDR = 1.37e−15) and the coagulation system (FRD = 8.83e−09) (Fig. 4 and Appendix C). Nevertheless, only part of these proteins cohort may have significance by results of the semi-quantitative analysis. Alterations of the observed proteins (Table 3) may follow an increased risk of clots formation and platelet aggregation. *SERPING1* can smooth this effect, but its abundance is undistinguished from the baseline (Table 3).

Unlike the M-series, in the C-series, the *PLG* correlated with *F2*, whereas *IGHM* correlated well enough with both proteins. Alterations in immunoglobulins is expected since the immune response suppressed during gestation, and the concentration of *IGHM* should be decreased^66^. However, we detected the increased abundance of *IGHM*, and the most pronounced elevation was indicated in groups of GDM positive patients with the affected newborns (G04P and G04P, Table 3). Recently, raising the concentration of *IGHM* has already been reported for patients with GDM^42^. Elevation of *IGHM* and depression of *IGHG*s has been suggested as a response to the growing blood glucose levels that can adversely affect the production of immunoglobulins^42,67^. The between-group quantitative distribution of *PLG*, *F2* and *IGHM* fostered to conclude whether all three markers are being above the baseline (control group), it seems possible to consider their combination as a predictor of possible DF predominantly for GDM positive patients. However, if both *PLG* and *F2* are below the baseline level, this may differentiate the obesity group (G06P, Table 3).

Complement factor *C7* was the only protein that was omitted from the interactome with the coagulation system. (Table 3). It is a part of the alternative path of compliment system together with overrepresented *CFB* and *CFH* (Table 3, Fig. 4) together with *C1QC* and *C1S* subcomponents. The role of *CFH* and *CFB* in developing insulin resistance^34, 36^ has been discussed while reviewing the M-series population. The personal significance of *CFB* was poorly reflected in our study since its alterations were not sufficient in contrast to the significantly raised *CFH* in groups with GDM fraught with DF (Table 3). In contrast, *C7* increased throughout study groups aside from the obesity group (G06P) where the protein level dropped down. This observation comes at odds with reported increased expression of C7 and defining it as a marker of obesity^68^ however, the research was provided on the omental adipose tissue.

In summary, changing the level of complement factors is not obviously associated with the diabetic condition and its dire consequences during gestation because immune response and cytokines activity are substantially affected during pregnancy^42,66,67^. But their combination with different types of immunoglobulins and selected proteins of hemostasis can be helpful for prospective evaluation of possible DF in patients with diabetes mellitus (Table 3).

#### Increased AFM under conditions of insufficient lipids transport and oxidative stress

The importance of *CP* has already been discussed in the context of the M-series. In the C-series, *CP* was upregulated in all groups too, thus, supporting its indication for the definition of GDM, T1DM, or T2DM. It was higher in groups associated with DF (Table 3), but in combination with apolipoproteins (*APOB*, *APOC1*, *APOC3*), *CP* can be used for sensitive recognizing of obese patients (group G06P, Tables 2 and 3) due to group-specific lowering. Since *CP* is non-transportation across the placenta^69^, its circulation in the cord blood was assumingly of fetal origination. The same can be supposed for *AGT* that can hardly be accounted as unambiguous indicator, but rather in proper combination with *CP*, *F2*, and *PLG* it can help reveal a risk DF.

The interactome analysis disclosed a core of proteins surrounding *CP*, among which the leading members were *AFM* and *CFH* (Fig. 4). Expression analysis showed a weak co-expression level between *AFM* and *CP* despite strong direct functional interaction. The *AFM* transports various hydrophobic molecules and carries vitamin-E under conditions of deficient or suppressed lipoprotein transport system and is involved in multiple pathologies, including diabetes mellitus^70^. It has recently been demonstrated that the *AFM* level was significantly lower in women with uncomplicated pregnancy compared to those with pre-eclampsia (1.2 folds change, p = 0.007)^68^. A correlation between *AFM* with GDM progression has also been reported^71^ whereupon an increased *AFM* expression was encouraged as an essential reference with high potency for pregnancy-related disorders^68,71^.

Our results demonstrated an elevation of *AFM* in all groups with GDM, especially in those with DF (G02P, G04P, G07P, and G09P, Table 3 and Fig. 4). This observation is agreed with AFM's known role in oxidative stress and, consequently, in the deployment of insulin resistance^72^. However, from our perspective, the co-occurred changes within the core of *AFM*, *CP*, and *CFH* were a secondary response to insulin resistance and might be caused by other dependent reasons. In particular, alterations of *CP* level can be explained by growing estrogen level in pregnancy^37^ or due to immune response and reflected as an acute inflammation protein. In turn, elevated levels of *AFM* may reflect the activation of compensatory mechanisms for the transport of lipids and lipophilic ligands related to impartments of glucose metabolism^69,73^. At once, co-expression with *CFH* cytokine reflects modulation of pro-inflammatory state and the impact on the remodeling of extracellular matrix caused by impairments in lipid metabolism and progressing insulin resistance^45,46^.

#### Chronic inflammation disrupts fibrinolysis

The main protein interacting with *AFM* is *HRG* (PPI = 0.683, Appendix C). Its most important functional feature is regulating immunoglobulins clearance and preventing their aggregation (Fig. 4)^74^. There is evidence stressing about the concentration of *HRG* in cord blood samples of premature newborns^75^. It was assumed that the increased *HRG* could be caused by the enhanced clearance of immune complexes and deploying an inflammatory reaction. In this case, *HRG* aggravates hemostasis impairments because *HRG* retards fibrinolysis by interfering with *PLG* for binding to fibrin^74,75^ (Fig. 4). This is especially underlined in patients with affected newborns since these groups (G02, G04, G07, and G09) demonstrated the most pronounced alterations in *PLG*, *F2*, complement factors, and immunoglobulins as has been discussed before (Tables 2 and 3).

Hence, monitoring *HRG* and *AFM* can be a valuable indicator for elucidating the risk of DF in patients with diabetes mellitus regardless of its etiology. Both proteins were characterized increasingly altered elevation in GDM positive groups with antenatal complications (G02P and G04P) while in T2DM and T1DM (G07P and G09P these proteins altered in a counter wise manner (Table 3). Moreover, considering that the increased *PLG* places GDM patients to a higher frequency of premature birth^75^ and its correlation with *HRG*, both proteins can be assumed as auxiliary markers for the risk of DF, specifically in GDM positive patients.

The tight functional junction of *ORM2* with the immune system and cluster of *AFM*, *CP*, and *HRG* attracted a particular interest. Together, these proteins form a core of localized in the extracellular space, and their biological activities are focused on the transport of various ligands (Fig. 4). The association of *ORM2* with insulin resistance in T2DM was earlier reported. The increased concentration of *ORM2* is associated with the chronic inflammation of peripheral tissue, which is typical in diabetes mellitus^76^. Here, we expectedly observed a manifested increase of *ORM2* in patients with GDM and T2DM due to chronic inflammation following exciting stimulation of the immune response. However, the differentiation of patients with T2DM and DF from patients with GDM is almost impossible due to overlapping fold change ranges. Nevertheless, generally, the risk of DF is lower if the relative abundance of *ORM2* significantly exceeds the baseline (Table 3), which may sufficiently support lipids transport and maintain the integrity of extracellular matrix in an acute inflammatory condition.

## Conclusion

We identified plenty of indicators for diabetes mellitus during gestation that can uncover the exact association of diabetic condition with the impaired fetal growth and development and impact on the morphological development. Obtained results demonstrate that T2DM and GDM are closely related conditions while T1DM is tracked separately, apparently, due to different mechanisms of the T1DM pathogenesis. Our data emphasize the close interaction between lipids metabolism, immune response, and the integrity of the extracellular architecture, which leads to an imbalance between hemostasis and immune response as the hallmark of diabetic conditions.

The specific interplay of the diabetes-causing biological processes during gestation is caused by both the progressing insulin resistance and estrogens' overproduction. We found that most of the identified and quantified proteins are shared between peripheral and cord blood samples, probably, due to the ability to cross the placental barrier.

It seems on the surface that the peripheral proteome of patients with diabetic condition reflects vigorously ongoing plasm-tissue lipids transport mediated, mainly, by the enhanced activity of PPAR and LXR receptors. Our findings improved the damage-centered hypothesis that is generally inspected for diabetic conditions designated by the associated turnover of Fe^3+^ transporters and the boosted production of ROS and RNS. On the proteome level, we also trapped the link between oxidative stress disrupted insulin clearance, specifically articulated in those patients who gave birth to the affected newborns.

Some non-transportation proteins were increased in the umbilical blood; thus, their presence is assumed to be of fetal origination and underlines that fetal growth and development are directly affected by the changes in maternal circulation through the joint mechanisms and signaling pathways. On the level of proteome changes, the impact was vastly observed for proteins responsible for remodeling of matrix architecture critical for the regular morphogenesis and causing, in particular, the high risk of diabetic cardiomyopathy and depression of neuronal tube development in the growing fetus.

Obviously, the diabetic condition impacts homeostasis, which is especially fraught during gestation. Signs of the disturbed fibrinolysis have been identified in both peripheral and cord blood samples, meaning the escalated immune response and the increased risk of pre-eclampsia in the maternal system. On the other hand, this consequently decreases maternal–fetal transport and causes warnings for fetal morphogenesis.

Indeed, a further longitudinal investigation is needed to build-up the proteome-scaled image of diabetes mellitus and its antenatal complications. However, in this study, we utilized the systematic approach to draw attention to the pathophysiological processes traced on the proteomic level in the interconnected maternal–fetal system.

## Limitations

Limitations of this study include the small size of cohort under consideration and period of the observation that may affect gross variance of some parameters. This may weaken the application and significance of the conclusion. Although there are plenty of markers for distinguishing diabetes mellitus during pregnancy and its complication affected fetal growth and development, due care must be taken for their consideration. Despite the long history of clinical study and treatment strategy of diabetes mellitus, it is still a complex disease with many uncertain gaps in molecular mechanisms of pathogenesis. Burden of responsibility for curation of such patients in pregnancy is twice matter since diabetic condition influences fetal growth and may promote undesirable consequence in the postpartum period, requiring longitudinal study on a broad population. Obviously, there is no single and strong protein marker, but their proper combination may help to recognize dire consequences.

## Materials and methods

### Ethical considerations

Design of the study and use of human material was approved by the local research ethics committee of the N.E. Bauman 29th Clinical Hospital (Moscow) and accomplished in accordance with the WMA Declaration of Helsinki on Ethical Principles for Medical Research Involving Human Subjects. The study included patients who routinely observed in course of ongoing gestation and gave birth in the N.E. Bauman 29th Clinical Hospital of Moscow between April of and August of 2019, and all donors gave their written consent to participate in the study.

### Subjects

The total population comprised of 264 subjects. Participants for the GDM groups were selected based on the results of OGTT and were subdivided into subgroups of patients who gave birth to healthy (G01, n = 30, BMI = 25.14 ± 4.17 kg/m^2^; G03, n = 26, BMI = 24.28 ± 5.13 kg/m^2^) and those who gave birth to newborns affected by the diabetic fetopathy (G02, n = 25, BMI = 25.39 ± 4.97 kg/m^2^; G04, n = 29, BMI = 25.71 ± 4.87 kg/m^2^). Patients with T1DM (G07, n = 24, BMI = 28.12 ± 4.72 kg/m^2^; G08, n = 18, BMI = 27.33 ± 5.06 kg/m^2^) and T2DM (G09, n = 32, BMI = 28.39 ± 6.11 kg/m^2^; G10, n = 28, BMI = 29.12 ± 5.43 kg/m^2^) were recruited based on the clinical records of their previous history of diabetes mellitus and were stratified by the criterion of manifested diabetic fetopathy in the same way as has been performed for the GDM positive patients. The obese patients were enrolled from the patients who delivered healthy newborns and had a BMI exceeded 31 kg/m^2^ (G06, n = 22, BMI = 34.79 ± 3.12 kg/m^2^). Control group of patients comprised of subjects with an uncomplicated and had a previous history of uncomplicated pregnancy and gave birth to healthy newborns (G05, n = 30, BMI = 22.68 ± 3.25 kg/m^2^). Details on the study cohort are shown in Table 1.

#### Inclusion criteria for a diabetic condition

Gestational diabetes mellitus has been established between 23–28 of gestational weeks during routine clinical screening using a 75-g OGTT according to the recommendations of IADPSG (revision 2010)^77^ adopted by Russian Association of Obstetrician and Gynecologist (revision 2012)^78^ in the following way: the fasting glucose level should not exceed 5.8 mmol/L and patients whose OGTT in one hour was below 9.8 mmol/L and had no previous history of any type of diabetes mellitus in clinical records were considered as the normal glycemic study group. Patients whose OGTT in one hour exceeded 9.8 mmol/L were passed to complete the next 2-h of OGTT for a final establishing of GDM and were considered as GDM-positive if OGTT showed more than 8.5 mmol/L.

Depending on the manifestation of GDM during gestation, biochemical records of HbAc1, and progression of HOMA-IR, patients were treated with either dietary intervention (G01 and G02, Table 1) or by insulin therapy (G03 and G04, Table 1). Patients with insulin treatment strategy received one of the following short-acting medications (bolus) three times per day (at 8 a.m., 1 p.m., and 6 p.m.) before meals: Insuman Rapid GT or NoVo Rapid FlexPen at a dose of 7–14 IU per day (totally) during the second and the third trimesters.

The duration of diabetes mellitus in groups with T1DM was 20.6 ± 5.8 years from the first clinical record, while in groups with T2DM it was 8.6 ± 4.4 years from the very first clinical record of diabetic condition manifestation and.

During the initial screening and selection, patients with a history of cardiovascular disease, any chronic disease, autoimmune diseases, or inflammatory were excluded. During gestation, patients with pre-eclampsia conditions were also excluded from the study cohort. Details about groups under consideration are given in Table 1.

#### Diagnostic criteria for diabetic fetopathy

The instrumental method of fetal biometry was performed using an Acuson 128 XP4 ultrasound machine (Siemens Inc., Munich, Germany), equipped with a 3.5‐MHz probe. Signs of fetopathy were established between 22–35 gestational weeks age as described in^42^ according to the following criteria: discreet fetal growth until 30 weeks, enlargement of the abdomen along with glycemic values at the lower limit, excessive macrosomia until 35 weeks; primarily established polyhydramnios if other reasons except GDM were not established; hepatosplenomegaly. Patients were stratified according to the clinical manifestation of diabetic fetopathy confirmed by ultrasound examination and in the following postpartum examination based on the clinical assay and Apgar-1/5 scores.

### Reagents

All reagents for sample preparation, liquid chromatography, and mass spectrometry analysis were analytical or HPLC grade, and details for all reagents are available in Appendix D.

### Samples collection and handling

Peripheral venous blood (4–6 mL, labeled as M-series samples) was collected from the patients into EDTA-2K^+^ Vacutainer plasma tubes (BD, USA). Cord blood (typically 10–15 mL, labeled as C-series samples) was collected in citrate phosphate dextrose solution. Samples were handled according to the manufacturer's instructions and centrifuged at 4 °C and 1800×*g* for 10 min. Samples were prepared for the proteomic assay and included steps of alkylation and enzymatic digestion with trypsin. Details of the preparation procedure are given in the Appendix D.

### Liquid chromatography and mass spectrometry analysis

Peptides were separated using liquid chromatography on an Ultimate 3000 RSLC (Thermo Scientific, Rockford, IL, USA). Samples were loaded onto an enrichment column Acclaim Pepmap (5 mm × 0.3 mm, 5 µm) for 4 min at a flow rate of 15 µL/min in a mobile phase C (water with 3.5% acetonitrile supplied with 0.1% formic acid and 0.05% acetic acid) and were separated onto analytical column Acclaim Pepmap (75 µm × 150 mm, 1.8 µm) at a flow rate of 0.30 µL/min in a gradient of mobile phases A (water) and B (90% acetonitrile and 10% methanol) both supplied with 0.1% formic acid and 0.03% acetic acid.

Mass spectrometry analysis was performed on a high-resolution Orbitrap Fusion (Thermo Scientific, Rockford, IL, USA) mass spectrometer. Precursor ions with charge states from z = 2 + to z = 6 + were surveyed at a resolution of R = 60 K in a range of 425–1250 *m/z*. Fragment ions were obtained at HCD activation energy normalized to 27% (ramping ± 20%) and detected in an ultra-high field orbital mass analyzer at a resolution of R = 15 K. The complete details are available in Appendix D^79,80^.

### Data analysis

Data files were converted to fit-for-searching format using MSConvert (Proteome Wizard). Data were searched using X!Tandem search engine against the human database (Uniprot release 2019.08) with concatenated decoy sequences. Searching was accomplished with preset of enzyme specificity and fixed/variable modifications. Results were extracted at 1% of the FDR level based on summarized false discovery rates for PSM. Complete details for searching parameters are given in Appendix D.

### Statistics, functional annotation and pathways analysis

Bias-correction of intra- and intergroup comparisons were evaluated using Kendal's *tau* correlation tests. Significances of between-group frequencies were evaluated by Fisher's exact test at a p-value cut-off of p = 0.01. Analysis of categorical features was performed using the Kruskal–Wallis test, and differences were considered significant at a cut-off of p < 0.05. The significance of anthropometric and biochemical parameters between studied groups was evaluated at a cut-off of p < 0.05 using the Mann–Whitney test. Alterations in the measured circulating proteins were estimated based on the NSAF (Normalized Spectral Abundance Factor) values obtained after protein identification. The approach estimated the mean values for each comparison group, and a two-sample moderated *t*-test was used to infer a p-value. The reported p-values were corrected for false discovery rate (FDR) and tested against a confidence level threshold of p < 0.05. Heatmaps of differential proteomes were constructed by Heatmapper^81^. Gene ontology analysis was performed with the PANTHER (Released 20181113) annotation tool with the p-value threshold at p < 0.001^82^ using Bonferroni correction for multiple testing. Molecular pathways were extracted from the following databases: KEGG^83^, STRING (version 10.5) and Reactome (version 65 Released 20180612)^84,85^.

## Supplementary information

Supplementary Information 1.

Supplementary Information 2.

Supplementary Information 3.

Supplementary Information 4.

Supplementary Information 5.

## Abbreviations

GDM: Gestational diabetes mellitus
DF: Diabetic fetopathy
T1DM: Type 1 diabetes mellitus
T2DM: Type 2 diabetes mellitus
OGTT: Oral glucose tolerance test
PPAR: Peroxisome proliferator-activated receptors
RXR: Retinoid X receptors
RAAS: Renin–angiotensin–aldosterone system
NSAF: Normalized spectral abundance factor
WHO: World Health Organization
IDF: International Diabetes Federation
DAVID: Database for Annotation, Visualization and Integrated Discovery
KEGG: Kyoto Encyclopedia of Genes and Genomes
