# Supplementary material for: Molecular pathophysiology of diabetes mellitus during pregnancy with antenatal complications
Source: Sci Rep. 2020 Nov 12;10:19641. doi: 10.1038/s41598-020-76689-9 (PMC7665025; doi:10.1038/s41598-020-76689-9)
Supplement: Supplementary file 2 — Supplementary Information 2. [file 41598_2020_76689_MOESM2_ESM.pdf]

Appendix B

MOLECULAR PATHOPHYSIOLOGY OF DIABETES MELLITUS DURING PREGNANCY WITH ANTENATAL COMPLICATIONS

Arthur T. Kopylov, Olga Papysheva, Iveta Gribova, Galina Kotaysch, Lubov Kharitonova, Tatiana Mayatskaya, Ekaterina Sokerina, Anna L. Kaysheva, Sergey G. Morozov

Matrices for the intergroup correlation estimated by Kendall's-tau rank correlation test. Kendall's tau coefficient may vary between t = (-1 ; 1). Value of t<0 designates negative correlation and the more negative absolute value, the stronger is correlation. The same is applicable for value t>0 but in this case positive correlation is suspected.

M-series Kendal correlation (peripheral blood)

group	G01M	G02M	G03M	G04M	G06M	G07M	G08M	G09M	G10M
G01M	1	0.43271274	0.86502824	0.80706464	0.42185977	0.11502757	0.40473878	0.44085986	0.5181282
G02M	0.43271274	1	0.8089853	0.48944453	-0.2358831	0.11310493	-0.0910198	0.93799996	-0.4082044
G03M	0.86502824	0.8089853	1	0.81649089	0.12456182	0.16019095	0.22403641	0.77885911	0.06836243
G04M	0.80706464	0.48944453	0.81649089	1	0.08018864	0.5585034	-0.0132978	0.46634854	0.12136062
G05M	0.42185977	-0.2358831	0.12456182	0.08018864	1	-0.0243677	0.3831309	-0.3886437	0.77408267
G06M	0.11502757	0.11310493	0.16019095	0.5585034	-0.0243677	1	-0.7575509	-0.0152161	-0.1087558
G07M	0.40473878	-0.0910198	0.22403641	-0.0132978	0.3831309	-0.7575509	1	-0.0164453	0.41163951
G09M	0.44085986	0.93799996	0.77885911	0.46634854	-0.3886437	-0.0152161	-0.0164453	1	-0.3836516
G10M	0.5181282	-0.4082044	0.06836243	0.12136062	0.77408267	-0.1087558	0.41163951	-0.3836516	1

P-series Kendal correlation (umbilical blood)

group	G01P	G02P	G03P	G04P	G06P	G07P	G08P	G09P	G10P
G01P	1	-0.4657298	0.60102329	-0.108264	-0.2483921	0.15954913	0.1860045	0.20896549	0.30459755
G02P	-0.4657298	1	-0.3398916	0.64973924	-0.0891251	-0.5544985	0.17865542	0.2418543	-0.358151
G03P	0.60102329	-0.3398916	1	-0.1944345	0.03315548	-0.1303481	-0.0502364	-0.0225628	-0.0571636
G04P	-0.108264	0.64973924	-0.1944345	1	0.0330856	-0.4587875	0.71556434	0.61655071	0.02975786
G05P	-0.2483921	-0.0891251	0.03315548	0.0330856	1	0.17678872	0.03259387	0.0421901	-0.4706873
G06P	0.15954913	-0.5544985	-0.1303481	-0.4587875	0.17678872	1	-0.4069545	-0.2024025	0.38999654
G07P	0.1860045	0.17865542	-0.0502364	0.71556434	0.03259387	-0.4069545	1	0.89648489	0.17196658
G09P	0.20896549	0.2418543	-0.0225628	0.61655071	0.0421901	-0.2024025	0.89648489	1	0.23074871
G10P	0.30459755	-0.358151	-0.0571636	0.02975786	-0.4706873	0.38999654	0.17196658	0.23074871	1

Relative ratio of proteins shared between groups in the M-series (peripheral blood)

NAME	Protein name	G01M	G02M	G03M	G04M	G06M	G07M	G08M	G09M	G10M
P00450	Ceruloplasmin	0.579	0.500	0.549	0.559	0.409	0.625	0.453	0.529	0.411
P00734	Prothrombin	0.337	0.331	0.371	0.336	0.286	0.394	0.341	0.589	0.289
P00747	Plasminogen	0.318	0.263	0.218	0.309	0.180	0.344	0.226	0.271	0.333
P00751	Complement factor B	0.340	0.342	0.319	0.302	0.266	0.306	0.351	0.220	0.287
P01009	Alpha-1-antitrypsin	1.678	2.295	1.913	1.811	2.080	1.781	2.458	1.879	1.716
P01019	Angiotensinogen	0.947	0.842	0.975	1.163	0.622	0.824	0.550	0.732	0.740
P01023	Alpha-2-macroglobulin	0.430	0.442	0.445	0.458	0.515	0.417	0.453	0.374	0.431
P01024	Complement C3	0.377	0.360	0.369	0.382	0.358	0.372	0.354	0.400	0.358
P01042	Kininogen-1	0.375	0.319	0.331	0.423	0.399	0.541	0.329	0.432	0.290
P01619	Immunoglobulin kappa variable 3-20	2.575	3.078	2.632	2.463	2.657	1.959	2.053	3.143	1.895
P01834	Immunoglobulin kappa constant	10.768	7.991	10.446	9.571	9.013	7.916	8.088	8.752	9.920
P01857	Immunoglobulin heavy constant gamma 1	2.510	2.478	2.275	2.760	3.221	2.068	2.528	2.380	2.035
P02647	Apolipoprotein A-I	2.347	2.343	2.261	2.156	1.894	2.741	2.806	2.183	2.162
P02649	Apolipoprotein E	0.899	0.836	0.731	0.917	0.895	1.002	0.957	0.862	0.901
P02652	Apolipoprotein A-II	3.036	3.639	3.301	3.711	4.299	3.054	3.072	3.530	2.305
P02656	Apolipoprotein C-III	2.233	2.198	2.199	2.367	1.583	3.012	2.119	2.497	2.106
P02760	Protein AMBP	0.730	0.620	0.630	0.732	1.005	0.976	0.543	0.417	0.610
P02763	Alpha-1-acid glycoprotein 1	2.256	1.889	2.194	2.340	2.916	2.301	2.573	1.617	2.092
P02765	Alpha-2-HS-glycoprotein	1.023	1.144	1.163	1.164	1.540	1.128	1.047	1.316	1.118
P02768	Serum albumin	3.227	2.828	3.059	3.434	4.090	2.730	3.443	3.137	3.426
P02787	Serotransferrin	1.502	1.806	1.457	1.552	1.765	1.420	1.628	1.427	1.692
P02790	Hemopexin	0.960	0.882	0.968	1.006	0.992	1.004	1.151	0.893	0.661
P04004	Vitronectin	1.015	0.971	0.969	0.904	0.566	1.026	0.881	0.960	0.884
P04114	Apolipoprotein B-100	0.104	0.120	0.101	0.116	0.068	0.119	0.079	0.116	0.100
P04217	Alpha-1B-glycoprotein	1.295	1.289	1.252	1.256	1.137	1.234	1.350	1.233	1.102
P08603	Complement factor H	0.227	0.188	0.220	0.258	0.123	0.195	0.200	0.168	0.138
P19652	Alpha-1-acid glycoprotein 2	1.487	1.050	1.376	1.558	1.394	1.417	1.256	1.036	1.320
P19823	Inter-alpha-trypsin inhibitor heavy chain H2	0.345	0.284	0.299	0.293	0.338	0.263	0.435	0.280	0.304
P19827	Inter-alpha-trypsin inhibitor heavy chain H1	0.363	0.365	0.327	0.344	0.470	0.533	0.427	0.481	0.391
Q14624	Inter-alpha-trypsin inhibitor heavy chain H4	0.407	0.450	0.423	0.437	0.394	0.381	0.407	0.368	0.304

Relative ratio of proteins shared between groups in the C-series (umbilical blood)

NAME	Protein name	G01P	G02P	G03P	G04P	G06P	G07P	G08P	G09P	G10P
O95445	Apolipoprotein M	0.604	0.761	0.586	0.711	0.500	0.646	0.477	0.621	0.539
P00450	Ceruloplasmin	0.223	0.278	0.247	0.246	0.181	0.264	0.182	0.291	0.250
P00734	Prothrombin	0.230	0.280	0.245	0.274	0.177	0.204	0.216	0.240	0.217
P00747	Plasminogen	0.176	0.220	0.166	0.211	0.136	0.190	0.164	0.252	0.208
P00751	Complement factor B	0.181	0.215	0.217	0.212	0.175	0.205	0.174	0.208	0.164
P01008	Antithrombin-III	0.293	0.318	0.321	0.313	0.257	0.274	0.270	0.272	0.254
P01009	Alpha-1-antitrypsin	1.866	1.972	1.879	1.654	1.598	1.659	1.464	1.491	1.832
P01011	Alpha-1-antichymotrypsin	0.411	0.435	0.343	0.478	0.423	0.330	0.326	0.328	0.391
P01019	Angiotensinogen	0.620	0.740	0.695	0.507	0.477	0.562	0.517	0.676	0.572
P01023	Alpha-2-macroglobulin	0.441	0.499	0.522	0.469	0.470	0.447	0.422	0.387	0.452
P01024	Complement C3	0.236	0.251	0.236	0.227	0.194	0.236	0.215	0.216	0.186
P01031	Complement C5	0.073	0.080	0.072	0.075	0.056	0.070	0.065	0.066	0.066
P01042	Kininogen-1	0.191	0.259	0.229	0.244	0.191	0.182	0.226	0.260	0.213
P01619	Immunoglobulin kappa variable 3-20	1.456	1.413	1.435	1.286	1.577	1.705	2.039	2.059	1.660
P01700	Immunoglobulin lambda variable 1-47	1.082	0.946	0.945	1.129	1.315	0.716	1.248	0.931	0.792
P01834	Immunoglobulin kappa constant	8.698	6.496	7.388	7.535	8.270	7.769	7.855	7.167	5.759
P01857	Immunoglobulin heavy constant gamma 1	2.938	2.665	2.960	2.887	3.185	2.885	2.372	2.758	3.059
P01859	Immunoglobulin heavy constant gamma 2	1.254	1.528	1.564	1.263	1.234	1.331	1.512	1.114	1.326
P01860	Immunoglobulin heavy constant gamma 3	0.562	0.506	0.572	0.624	0.599	0.528	0.569	0.484	0.457
P01861	Immunoglobulin heavy constant gamma 4	0.756	0.684	0.759	0.690	0.753	0.559	0.879	0.799	0.643
P01871	Immunoglobulin heavy constant mu	0.350	0.388	0.360	0.451	0.496	0.331	0.283	0.455	0.320
P02647	Apolipoprotein A-I	1.773	2.284	2.106	1.988	2.201	1.727	1.591	1.666	1.737
P02649	Apolipoprotein E	0.537	0.595	0.545	0.552	0.689	0.558	0.601	0.594	0.532
P02652	Apolipoprotein A-II	2.802	2.840	2.674	3.017	3.346	2.573	2.914	2.519	3.604
P02655	Apolipoprotein C-II	1.124	0.922	1.101	1.046	0.781	0.891	1.141	1.139	1.035
P02656	Apolipoprotein C-III	1.085	0.936	1.293	1.030	1.010	1.358	1.271	1.088	1.147
P02747	Complement C1q subcomponent subunit C	0.474	0.526	0.453	0.359	0.349	0.642	0.330	0.445	0.405
P02749	Beta-2-glycoprotein 1	0.492	0.495	0.504	0.410	0.385	0.427	0.409	0.509	0.406
P02751	Fibronectin	0.078	0.104	0.085	0.081	0.045	0.082	0.063	0.070	0.055
P02753	Retinol-binding protein 4	0.622	0.530	0.565	0.534	0.613	0.569	0.507	0.496	0.377

P02760	Protein AMBP	0.469	0.616	0.501	0.518	0.508	0.574	0.578	0.685	0.414
P02763	Alpha-1-acid glycoprotein 1	1.160	1.224	1.178	1.171	1.492	1.244	1.007	1.033	1.180
P02765	Alpha-2-HS-glycoprotein	1.749	1.990	1.753	1.766	1.696	1.775	1.515	2.127	1.774
P02766	Transthyretin	0.668	0.497	0.601	0.841	1.216	0.831	0.719	0.587	0.771
P02768	Serum albumin	2.565	2.394	2.754	2.861	3.684	2.290	2.816	2.404	2.852
P02774	Vitamin D-binding protein	0.453	0.453	0.414	0.439	0.439	0.438	0.445	0.492	0.575
P02775	Platelet basic protein	0.914	1.096	1.111	0.974	0.861	0.993	1.111	1.219	0.822
P02776	Platelet factor 4	0.980	0.963	0.980	0.931	0.983	1.180	0.611	1.002	0.725
P02787	Serotransferrin	1.017	1.033	0.900	0.951	0.962	0.798	0.893	1.111	0.919
P02790	Hemopexin	0.391	0.405	0.463	0.401	0.387	0.409	0.452	0.425	0.450
P04004	Vitronectin	0.386	0.480	0.372	0.442	0.396	0.349	0.500	0.506	0.430
P04114	Apolipoprotein B-100	0.049	0.053	0.052	0.052	0.036	0.042	0.047	0.057	0.056
P04196	Histidine-rich glycoprotein	0.197	0.195	0.221	0.190	0.137	0.144	0.212	0.177	0.117
P04217	Alpha-1B-glycoprotein	0.580	0.605	0.587	0.577	0.531	0.595	0.458	0.662	0.611
P04433	Immunoglobulin kappa variable 3-11	1.346	1.344	1.244	1.204	0.837	1.166	1.286	1.496	1.028
P05090	Apolipoprotein D	0.633	0.744	0.577	0.679	0.541	0.637	0.478	0.699	0.448
P05155	Plasma protease C1 inhibitor	0.439	0.439	0.415	0.409	0.401	0.414	0.368	0.557	0.435
P05543	Thyroxine-binding globulin	0.233	0.276	0.242	0.222	0.196	0.253	0.160	0.200	0.148
P05546	Heparin cofactor 2	0.209	0.227	0.231	0.249	0.160	0.216	0.205	0.230	0.220
P06312	Immunoglobulin kappa variable 4-1	1.912	2.202	1.769	1.988	2.281	1.471	1.428	1.831	2.054
P06396	Gelsolin	0.224	0.239	0.233	0.217	0.191	0.238	0.183	0.254	0.153
P06727	Apolipoprotein A-IV	0.410	0.380	0.350	0.353	0.346	0.338	0.321	0.445	0.335
P08185	Corticosteroid-binding globulin	0.242	0.292	0.288	0.288	0.138	0.221	0.201	0.268	0.224
P08603	Complement factor H	0.169	0.201	0.189	0.183	0.144	0.167	0.161	0.158	0.169
P08697	Alpha-2-antiplasmin	0.473	0.596	0.432	0.386	0.368	0.354	0.440	0.416	0.340
P09871	Complement C1s subcomponent	0.126	0.158	0.133	0.147	0.103	0.144	0.095	0.133	0.100
P0C0L5	Complement C4-B	0.102	0.117	0.108	0.106	0.079	0.099	0.098	0.111	0.093
P10643	Complement component C7	0.128	0.143	0.146	0.118	0.073	0.124	0.098	0.149	0.111
P10909	Clusterin	0.344	0.438	0.414	0.415	0.271	0.426	0.319	0.409	0.466
P13671	Complement component C6	0.041	0.065	0.039	0.052	0.030	0.048	0.042	0.053	0.059
P15814	Immunoglobulin lambda-like polypeptide 1	0.319	0.328	0.297	0.261	0.279	0.283	0.325	0.272	0.422
P19652	Alpha-1-acid glycoprotein 2	0.865	0.958	1.061	0.939	0.783	0.974	0.641	0.851	0.631
P19823	Inter-alpha-trypsin inhibitor heavy chain H2	0.262	0.275	0.246	0.249	0.231	0.253	0.200	0.334	0.208
P19827	Inter-alpha-trypsin inhibitor heavy chain H1	0.260	0.266	0.275	0.265	0.239	0.270	0.280	0.392	0.241
P22792	Carboxypeptidase N subunit 2	0.143	0.139	0.173	0.183	0.126	0.167	0.129	0.133	0.278

P23142	Fibulin-1	0.106	0.104	0.116	0.153	0.085	0.129	0.055	0.121	0.094
P25311	Zinc-alpha-2-glycoprotein	0.558	0.541	0.543	0.581	0.499	0.507	0.454	0.667	0.648
P35542	Serum amyloid A-4 protein	0.456	0.594	0.490	0.483	0.434	0.298	0.551	0.714	0.496
P36955	Pigment epithelium-derived factor	0.204	0.242	0.222	0.194	0.170	0.190	0.175	0.289	0.174
P43652	Afamin	0.233	0.265	0.237	0.226	0.234	0.189	0.187	0.255	0.220
P51884	Lumican	0.477	0.477	0.501	0.464	0.439	0.525	0.353	0.481	0.462
P68871	Hemoglobin subunit beta	1.575	1.351	1.488	2.136	2.096	1.689	1.878	1.398	2.297
P69891	Hemoglobin subunit gamma-1	1.234	1.262	1.251	1.523	1.959	1.245	1.842	1.152	2.504
P69905	Hemoglobin subunit alpha	2.087	1.521	2.155	2.395	2.878	2.111	3.114	1.888	4.111
Q06033	Inter-alpha-trypsin inhibitor heavy chain H3	0.130	0.153	0.153	0.122	0.108	0.129	0.137	0.126	0.215
Q14624	Inter-alpha-trypsin inhibitor heavy chain H4	0.271	0.240	0.269	0.239	0.205	0.221	0.224	0.280	0.233
Q96PD5	N-acetylmuramoyl-L-alanine amidase	0.211	0.234	0.206	0.213	0.149	0.227	0.179	0.241	0.154
