# Supplementary material for: Molecular pathophysiology of diabetes mellitus during pregnancy with antenatal complications
Source: Sci Rep. 2020 Nov 12;10:19641. doi: 10.1038/s41598-020-76689-9 (PMC7665025; doi:10.1038/s41598-020-76689-9)
Supplement: Supplementary file 4 — Supplementary Information 4. [file 41598_2020_76689_MOESM4_ESM.pdf]

Appendix D

MOLECULAR PATHOPHYSIOLOGY OF DIABETES MELLITUS DURING PREGNANCY WITH ANTENATAL COMPLICATIONS

Arthur T. Kopylov, Olga Papysheva, Iveta Gribova, Galina Kotaysch, Lubov Kharitonova, Tatiana Mayatskaya, Ekaterina Sokerina, Anna L. Kaysheva, Sergey G. Morozov

This supplement is for the "Material and Methods" section with extra details concerning the Experimental Procedure and Data Analysis.

Reagents

Urea (99%) and formic acid (98%+) were obtained from Acros Organics (Geel, Belgium). Trifluoroacetic acid (99%, Reagent Plus®), triethylammonium bicarbonate (1 M solution), 4-vinylpyridine (95%), sodium deoxycholic acid (>97% titration) and ammonium acetate (BioXtra, >98%) were from Sigma (St. Louis, MO, USA). Acetonitrile (HPLC grade, filtered for 0.2 µm) was purchased from Fisher Chemical (Loughborough, UK). Acetic acid (EMSURE®, glacial, anhydrous for analysis) was from Merck (Darmstadt, Germany). TCEP-HCl (Tris(2-carboxyethyl) phosphine hydrochloride) was purchased from Pierce™ (Thermo Fisher, Rockford, IL, USA). Trypsin (sequencing grade modified) was supplied by Promega (Madison, WI, USA). Water (TOC<3 ppb, 18.2-18.5 mΩ*cm) was obtained from Milli-Q Integral 3 purification system, Millipore S.A.S (France).

Samples collection and handling

Venous blood samples (4-6 mL of maternal blood, labeled as M-series samples) were collected from the patients into EDTA-2K^+ Vacutainer plasma tubes (BD, USA). Human cord blood samples (typically 10-15 mL, labeled as C-series samples) of the same donors were collected in citrate phosphate dextrose solution. The blood samples were processed according to the manufacturer's instructions and centrifuged at 4°C and 2500 *g* for 10 min. The plasma supernatant was filtered through 0.22-µm cellulose-acetate filters (Whatman, NJ, USA) and stored at -80°C. Plasma samples in amount of 100 µg (typically 2-2.5 µL) were transferred into a clean tube, and denaturation solution (5 M urea, 1% deoxycholic acid sodium salt, 15% acetonitrile, 100 mM phosphate buffer pH 6.3, 300 mM sodium chloride, 20 mM TCEP) in a volume of 20 µL was added to a final concentration of total protein close to 10 mg/mL. Samples were heated for 10 minutes at 60°C, chilled at room temperature, and supplemented with 25 µL of 15 mM 2-iodoacetamide in 50 mM triethylammonium bicarbonate. The alkylation reaction was incubated for 30 minutes at ambient temperature following dilution up to 120 µL in 50 mM triethylammonium bicarbonate to decrease the final concentration of denaturation buffer compounds and achieve a final protein concentration close to 1 mg/mL. Trypsin in the amount of 1 µg (5 µL of 200 ng/mL) was added to the samples and incubated for 3 hours at 38°C, following the addition of the next aliquot of enzyme (1 µg) and incubation for an additional 3 hours. After the completion of digestion, 10 µL of 10% formic acid was added to the samples. The samples were centrifuged for 10 minutes at 10°C at 12,000 *g* to sediment insoluble deoxycholic acid. The supernatant was quantitatively (90 µL) transferred into a clean tube.

Liquide chromatography

Peptides were separated using liquid chromatography on an Ultimate 3000 RSLC Nano (Thermo Scientific, Rockford, IL, USA) system equipped with 5 µL loop (loop flush out multiplier – 3). Samples were continually in autosampler thermostat at 6±1°C. Samples were drawn at 12 µL/min speed and injected into the loop at 15 µL/min. Samples were loaded onto an enrichment column Acclaim Pepmap® (5 mm x 0.3 mm, 300A pore size, 5 µm particle size) for 4 minutes at a flow rate of 15 µL/min in a mobile phase C (water with 3.5% acetonitrile supplied with 0.1% formic acid and 0.05% acetic acid, pH = 2.75 at t = 21°C). Peptides were washed out from the enrichment column and separated onto analytical column Acclaim Pepmap® (75 µm x 150 mm, 1.8 µm particle size, 60A pore size) at a flow rate of 0.30 µL/min in a gradient of mobile phase A (water, pH 2.67 at t = 20.7°C) and mobile phase B (90% acetonitrile and 10% methanol) both supplied with 0.1% formic acid and 0.03% acetic acid. Dynamic flow rate from 0.30 to 0.45 µL/min within 1.25 minutes were applied for column washing in mobile phase B for 6 minutes.

Mass spectrometry

Mass spectrometry analysis was performed on a high-resolution Orbitrap Fusion (Thermo Scientific, Rockford, IL, USA) mass spectrometer. The instrument was equipped by NSI ion source and operated in a positive ionization mode using three-segmental data-dependent survey. The electrodynamic S-lens RF was adjustment at 70% and capillary vaporizing temperature set to 280°C. Precursor ions with charge states from z=2+ to z=6+ were surveyed at a resolution of R=60K in a range of 425 – 1250 m/z. Ions were isolated using quadrupole isolation width ±1.5 Th and offset of +0.5 Th. The maximum integration time was 15 ms, or AGC set to 4e5 ions. The tandem MS/MS acquisition of precursor ions was triggered at a level of 45% of chromatography peak apex (pre-set average FWHM was 24 s) if minimal SNR was at least 1500 counts. Fragment ions were obtained at HCD activation energy normalized to 27% ramped within ±20% and detected in an ultra-high field orbital mass analyzer at a resolution of R=15K. Ions were accumulated for a maximum integration time of 47 ms, or AGC set to 5e4 ions. Active dynamic exclusion was triggered for 180 sec if 6 repeats within 4 sec was recorded for all parallel charge states of the injected precursor ions. The complete one duty cycle time was 4 sec.

Data analysis

Vendors original data files (original raw-format) were converted to fit-for-searching mgf-format using MSConvert (Proteome Wizard). Data were searched using X!Tandem search engine against taxonomy-specific database (Human, Uniprot release 2018.08) enriched with an equal number of decoy reverse sequences. The pre-installed search parameters considered trypsin as a digestion enzyme with 2 maximum internal missed cleavages. The allowed charge states were from z = 2+ to z = 6+ with precursor tolerance of ±5 ppm and fragment ions tolerance of ±0.01 Da. Variable modification used for searching and discovery were deamidation of Q/E, methionine single oxidation, and 4-hydroxyproline. The fixed modification was pyridilethylation by 4-vinylpyridine. Results were extracted at no more than 1% of FDR level based on summarized false discovery rate for PSM, peptides and proteins with dynamic correction of the recovered sequence results with molecular weight of the canonical protein sequence.

Pilot statistical analysis and exclusion of outliers

In the overwhelming majority, biological systems are represented by complex arrays with multiple co-influencing effects which not always can be determined precisely or numerically. To perceive samples under the study to an aligned condition with a probability density distribution close to normal, we used descriptive statistics on the pilot steps (Appendix A). Measured ranges can be wide enough for the majority of proteins and may cover up to two orders of magnitude that causes the need for additional corrective actions in statistical analysis.

The error assessments for each protein of study group varied somewhat depending on the group of study. Some subjects within every group were excluded as outliers based on the significant bias from the baseline (Appendix A). There were some cases of extreme statistical values that still meet statistical confidence. Among them, SERPINA1 was characterized by negative kurtosis E=-(0.699-0.917) indicating flat-top distribution below the mean value (Appendix A) while asymmetry As=0.484-0.601 confirms shift of distribution to a larger than the mean value. If the relative concentration of SERPINA1. The protein AGT is another example of more prominent negative kurtosis E=-(1.342-1.411), while the asymmetry coefficient also has a negative value (A=-0.252). Both indicators indirectly argue about disturbances in the RAAS regulation of electrolyte homeostasis and hormones-mediated regulation of blood pressure, which is, however, usual during pregnancy. The opposite situation was observed for protein A2M, where kurtosis was represented by the maximum value over proteins population (E=+5.739), which indicates a wide and significant exceeding of the measured values relative normal distribution. A positive As=1.559 argues that about 69% of all numerical measured values were higher than the mean by 73%-87%. This is consistent with the well-known data on the reasons of A2M fluctuations associated with embryogenesis during pregnancy and wit long-term diabetes mellitus.

The dispersion for each identified and measured protein was within acceptable limits and the confidence interval of the mean values is within 4.9%–16.8%. The kurtosis value in most cases

is not equal to zero, however, asymmetry exhibits greater inter-group heterogeneity for the same protein. Complete data are represented in Appendix A.

Featured cases for correlation analysis in study cohorts

Distortions in the primary statistics may be due to many reasons: hidden patholophysiological processes, subclinical infections and peculiarities of the gestation course. Next step, patients within each individual group were analyzed by rank correlation analysis to evaluate the convergence of distribution within individual group. Spearman rank correlation was used for identification of discrepancies within a group, and revealing signs of discrimination (classification) between groups since, as expected, patients within individual group should have a similar features distribution. Complete proteins distribution across study groups is provided in the Appendix A.

In some groups (G03M, G04M, G01P) the correlation between subjects was accounted from 0.29 to 0.33 and 0.41. In the G04M group, only one patient (number 80) turned out to be the least relevant, and the minimal correlation was presented with patients 50 and 06 (r=0.69 and r=0.67, respectively). Group G01P was attributed by seldom correlation reached in a pair of patients 69 and 26 (r=0.59). It was sssumed other associated diseases that had a rather strong influence on the proteomic profile of each irrelevant patient and, accordingly, the constitutive profile of the study groups.

We observed that typical reason of intragroup biases of correlation was caused by a reduced concentration of a variety of proteins: F2, CFB, CP, IGHM, APOC3, IGKC. Since these proteins are represented in LDL and HDL transport, copper transfer and acute, it could be suggested that such patient has a chronic hepatopathy. However, the relative abundances of A2M and haptoglobin were only slightly out of range: 0.386 vice 0.431 and 0.951 vice 1.135 in relative units, respectively. On the other hand, a decrease in the relative abundance of transport proteins and immune proteins might indicate a systemic disorder related to lipid and electrolyte metabolism, which in turn can lead to nephropathy and autoimmune response.

In the groups G02M, G07M, G03P, G04P some patients were excluded from the further consideration by reason of the increased relative abundance of certain proteins among which the most striking included PON1, CFH, HRG, JCHAIN, A1BG, TF and IGKV3-20. The pattern of these proteins in the excluded patients was approximately twice exceeding of the mean pattern within these groups. Eventually, after the correlation analysis the assayed groups were treated for systematization and alignment by proteins abundance distribution.

The intergroup rank correlation by Kendal’s analysis allows to compare all the groups of both series (M and P) within compiled symmetric matrix. It was necessary to elect commonly identified proteins that constitute dynamic proteome across all groups within each series. The resulting proteome patterns were 30 proteins in the M-series and 77 proteins in the C-series (Appendix B). The average distance between patients within same study group of M-series was d^2=0.189, and in the C-series the average distance between patients within same study group was d^2=0.204, which reflects effectiveness of the previous correction action. The data of intergroup correlation analysis were tested by the paired two-tailed F-test for given significance level of a=0.01.

In the M-series, the maximum correlation was observed in the cluster of G01M-G04M groups, where the Kendal’s coefficient was t=0.80–0.86, and between G02M and G09M (t=0.94) (Figure 1). Patients with GDM can be reliably distinguished from the control group and patients with T1DM and T2DM. Group G09M was significantly varying by minimal correlation with control G05M-G07M where correlation was negative (t=-0.10) (Figure 1). Compilation of these features thereby specifies patients with GDM, and notable groups of obesity (G06M) was minimally correlated with all other patients (Appendix B).

In the C-series, group G03P was the most explicit example of negative correlation toward G02P, G04P, G06P-G10P (Figure 1). The maximum Kendal’s correlation cluster was being reached between groups G04P, G06P, G07P, G09P (t=0.89-0.64) while the strongest negative correlation was reached between G06, G)7P, G02P, G04P (t=-(0.56-0.40)). Complete correlation data are shown in Appendix B.
